# Prevalence estimates of *Opisthorchis viverrini* and *Clonorchis sinensis* infection in the Greater Mekong subregion: a systematic review and meta-analysis

**DOI:** 10.1186/s40249-024-01201-8

**Published:** 2024-05-08

**Authors:** Pornphutthachat Sota, Morsid Andityas, Manas Kotepui, Banchob Sripa

**Affiliations:** 1 WHO Collaborating Centre for Research and Control of Opisthorchiasis (Southeast Asian Liver Fluke Disease), Tropical Disease Research Center, Khon Kaen University, Khon Kaen, Thailand; 2 Faculty of Veterinary Medicine, Khon Kaen University, Khon Kaen, Thailand; 3 Department of Bioresources Technology and Veterinary, Veterinary Technology Study Program, Vocational College, Universitas Gadjah Mada, Depok, Indonesia; 4 School of Allied Health Sciences, Walailak University, Nakhon Si Thammarat, Thailand; 5 Department of Tropical Medicine, Faculty of Medicine, Khon Kaen University, Khon Kaen, 40002 Thailand; 6 Medical Technology Program, Faculty of Science, Nakhon Phanom University, Nakhon Phanom, Thailand

**Keywords:** *Opisthorchis viverrini*, *Clonorchis sinensis*, Great Mekong subregion, Human, Meta-analysis

## Abstract

**Background:**

Opisthorchiasis and clonorchiasis, caused by *Opisthorchis viverrini* and *Clonorchis sinensis*, respectively, are significant yet neglected foodborne trematodiases in the Great Mekong Subregion (GMS). Despite the reporting of the prevalence of these human liver flukes in the region over the past decades, there has been a lack of a comprehensive and systematic consolidation of this data. Therefore, we aimed to conduct a thorough systematic review and meta-analysis to synthesize and analyze time-trend prevalence estimates of both *O. viverrini* and *C. sinensis* across the GMS for the past 30 years.

**Methods:**

This study undertakes a systematic review using a comprehensive search for published articles in PubMed, EMBASE, Scopus, Cochrane and Thai Journal Online databases until early 2023. The pooled prevalence of *O. viverrini* and *C. sinensis* infection was analyzed through a random-effects meta-analysis, with meta-regression analysis used to quantify associations with study characteristics. Sub-group analysis was conducted, whenever comparison data were available, to assess the risk of *O. viverrini* and *C. sinensis* infection in each GMS country. Heterogeneity among studies was assessed using the Q statistic and quantified by using the *I* ^2^ Index.

**Results:**

From a total of 2997 articles, 155 articles comprising 218 datasets and 751,108 participants were included for review. The GMS prevalence of *O. viverrini* was 21.11% [45,083/260,237; 95% confidence interval (*CI*): 17.74–24.47%]. Pooled prevalence estimates were highly observed in Laos (34.06%, 95% *CI*: 26.85–41.26%), followed by Thailand (18.19%, 95% *CI*: 13.86–22.51%), and Cambodia (10.48%, 95% *CI*: 5.52–15.45%). Myanmar and Vietnam had limited data sources for calculation. *Clonorchis sinensis* infection in GMS was 25.33% (95% *CI*: 18.32–32.34%), with Guangxi, China, exhibiting the highest prevalence rates at 26.89% (95% *CI*: 18.34–35.43%), while Vietnam had a prevalence rate of 20.30% (95% *CI*: 9.13–31.47%). *O. viverrini* prevalence decreased significantly over time, whereas *C. sinensis* infection appeared to be stable consistently over time in both China and Vietnam.

**Conclusions:**

This comprehensive study, drawing from the largest datasets to date, offers an in-depth systematic prevalence review of human liver flukes in the Greater Mekong Subregion. It underscores the imperative for systematic surveillance, data collection, and the implementation of intervention and control measures for these infectious diseases of poverty.

**Graphical Abstract:**

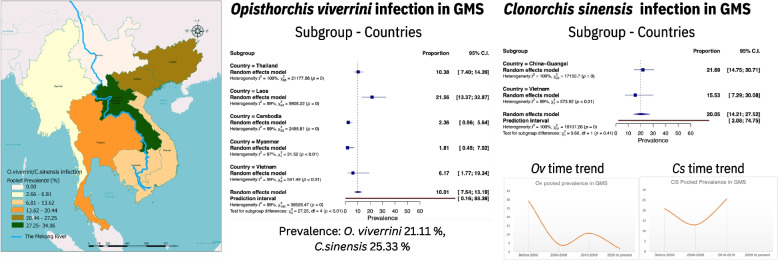

**Supplementary Information:**

The online version contains supplementary material available at 10.1186/s40249-024-01201-8.

## Background

Foodborne trematodiases caused by human liver fluke infections, *Opisthorchis viverrini* and *Clonorchis sinensis*, represent major neglected tropical diseases (NTDs) in the Great Mekong Subregion (GMS), with 15–18 million reported infections [1, 2]. *O. viverrini* is endemic in Thailand, Lao People's Democratic Republic (Lao PDR), Cambodia, the central and southern parts of Vietnam, and Myanmar, while *C. sinensis* is endemic in northern Vietnam and the Guangxi-Zhuang Autonomous Region of China. These liver fluke infections are associated with various hepatobiliary morbidities and mortalities, including cholangitis, gallstones, periductal fibrosis, and cholangiocarcinoma (CCA), a fatal bile duct cancer [3]. Both human epidemiological studies and experimental evidence implicate liver fluke infections in the development of CCA [3, 4]. Notably, both *O. viverrini* and *C. sinensis* are classified as Group 1 Biological Carcinogens by the World Health Organization International Agency for Research on Cancer [4]. Thailand has reported the highest incidence of this bile duct cancer globally [5]. CCA imposes a substantial disease burden in Thailand and the GMS. Phoncharoen et al. (2018) reported a high disability-adjusted life years (DALYs) for CCA attributable to liver fluke infection, ranging between 70,745 and 138,221 [6].

Liver fluke infections not only cause diseases but also contribute to the socioeconomic burden of affected individuals and households due to the loss of family heads and the costs associated with treatment or surgery [7]. The burden of disease is closely tied to the prevalence of liver fluke infections. However, these infections remain underreported in many GMS countries, particularly in Cambodia, Myanmar, and even in China [2]. Despite various reports on the prevalence of *O. viverrini* and *C. sinensis* in the GMS over past decades, there has been a lack of a comprehensive and systematic consolidation of this data. This gap hinders a full understanding of the current prevalence and trends of these significant yet neglected foodborne trematodiases. Consequently, we conducted an in-depth systematic review and meta-analysis of the prevalence of *O. viverrini* and *C. sinensis* in all GMS countries, including Thailand, Lao PDR, Cambodia, Myanmar, Vietnam, and Guangxi Zhuang Autonomous Region and Yunnan Province of China, over time. Our aim is to understand the time trend of prevalence in each GMS country over the past three decades. These data will provide critical updates on the disease burden of human liver flukes in the GMS, and the information may prove useful for prioritizing and adjusting public health interventions for better disease management in the GMS.

## Methods

### Protocol and registration

This study followed the Preferred Reporting Items for Systematic review and Meta-Analyses guidelines [8]. The review was registered at the International Prospective Register of Systematic Reviews website (CRD42023397229).

### Definitions

GMS is a trans-national region of the Mekong River basin in Southeast Asia. It comprise the six Asian countries of Lao PDR, Myanmar (Burma), Cambodia, Thailand, Vietnam, and China (Yunnan Province and the Guangxi Zhuang Autonomous Region) [9].

### Review question

The review question followed the Condition, Context, Population (CoCoPop) approach [10]. The approach considers the following components: *Co (Condition)*: the presence of human liver flukes (*O. viverrini* and *C. sinensis*). Co (Context): Studies conducted within the GMS [Cambodia, Lao People's Democratic Republic (Lao PDR), Myanmar, Thailand, Vietnam, and the People's Republic of China, specifically Yunnan Province and Guangxi Zhuang Autonomous Region] [2], encompassing various settings such as rural communities, hospitals, or other relevant environments where *O. viverrini* and *C. sinensis* infections were examined. The review question of this study was “What is the prevalence estimate of *O. viverrini* and *C. sinensis* infection in the GMS?

### Search strategy

For this systematic review and meta-analysis, we conducted a comprehensive search for epidemiological studies across five biomedical databases: PubMed, EMBASE, Scopus, Cochrane and Thai Journal Online, spanning from all relevant records published up to February 9, 2023. Additional information, when necessary, will be sought by contacting the authors of relevant studies. The search employed the following terms: (("liver fluke" OR "*Opisthorchis viverrini*" OR "opisthorchiasis" OR "*Clonorchis sinensis*" OR "clonorchiasis") AND ("prevalence" OR "epidemiology" OR "incidence" OR "survey")), with no language restrictions. The search strategy was tailored for each database to ensure inclusivity (see Additional file 1: Table S1 for detailed strategies).

### Eligibility criteria and study selection

The inclusion criteria comprised cross-sectional studies, interventional studies, and surveys identifying *O. viverrini* or *C. sinensis* among humans in the GMS. Exclusion criteria were applied to studies with insufficient data for extraction, meeting any of the following conditions: research without full-text access, reviews, systematic reviews, In vitro studies, conference proceedings, case–control studies, overlapped datasets with other publications, studies conducted in other GMS regions or countries, sample size less than 30 individuals, case reports or series, letters, commentaries. To ensure the reliability and methodological rigor required of the meta-analysis, a minimum sample size criterion of 30 + individuals was guided by methodological considerations aimed at minimizing between-study heterogeneity as suggested previously [11]. Studies with fewer than 30 participants can disproportionately influence meta-analysis outcomes due to their inherent variability and potential biases (small-study effect).

Articles were imported into Rayyan Qatar Computing Research Institute (QCRI) [12] from the databases, and duplicates were removed during the initial screening. Two researchers (PS and MA) independently reviewed titles and abstracts based on eligibility criteria. The electronic search generated titles and abstracts were the first to be checked. After reviewing full texts, studies not meeting inclusion requirements were excluded, with reasons documented. Disagreements between reviewers were resolved through consultation with a third reviewer (MK or BS).

### Data extraction

The data extraction process encompassed information on the first author, study procedures (design, sites, population characteristics, year of data collection, diagnostic tests utilized), and outcomes (number of cases, total population) (Additional files 2 and 3). Study sites referred to the countries and provinces where the research was conducted. The population studied was categorized into demographic subgroups, including children (aged 1 to 17), adults (aged 18 and older), and individuals of all ages across various populations.

Studies covering multiple locations, collecting data in different years, or examining diverse population groups often provided multiple prevalence estimates. In such cases, we considered all relevant estimates for analysis. When studies reported prevalence based on different diagnostic tests, and total positivity was indicated, we prioritized this value. Common tests such as formalin-ether concentration technique (FECT) or Kato-Katz were used when applicable. In instances where this was not explicitly stated, data corresponding to the diagnostic approach with the highest recorded prevalence were selected.

Data extraction was carried out independently by two investigators (PS, MA) using a standardized form in Microsoft Excel spreadsheets (Microsoft Corporation, Redmond, USA). Discrepancies between investigators were resolved through discussion among PS, MA, BS, and MK.

### Risk of bias and quality assessment

The risk of bias in the selected studies was evaluated utilizing the Joanna Briggs Institute (JBI) Critical Appraisal Tools designed for cross-sectional, case–control, cohort, and randomized controlled studies [10]. These tools comprehensively assess studies across nine categories. In adherence to the guidelines outlined for these tools, each study underwent classification based on its risk of bias, categorized as low (score: 7–9/9), moderate (4–6/9), or high risk of bias (3/9). Risk of bias was carried out independently by two investigators (PS, MA). Discrepancies between investigators were resolved through discussion with another investigator (MK).

### Data analysis

The data underwent analysis using the R software (version 4.3.1, R foundation for Statistical Computing, Vienna, Austria) with the Meta package [13]. To estimate the prevalence of *O. viverrini* and *C. sinensis* infection in each study, the number of test-positive cases was divided by the study population. A logit transformation of proportional data was performed following the methodology outlined by Lipsey and Wilson (2001) [14]. Utilizing a meta-analysis technique and a random-effects model with 95% confidence intervals, pooled estimates of the prevalence of *O. viverrini* and *C. sinensis* were derived [15]. The Q statistic [16] was employed to assess the heterogeneity of prevalence estimates among studies, and the *I* ^2^ Index [17] quantified this heterogeneity. Subgroup analyses were conducted on the research data to explore potential sources of heterogeneity, examining age groups, diagnostic methodologies, collection periods, and geographical regions.

Pooled prevalence estimates for individual infection in humans were obtained from relevant data from at least two studies, enhancing the robustness of the findings. Subgroup analyses for *O. viverrini* and *C. sinensis* were conducted for each country and time period to determine the prevalence of each liver fluke. Additionally, a meta-regression analysis was performed to elucidate trends in *O. viverrini* and *C. sinensis* infections in humans over time.

To visually represent estimates of *O. viverrini* and *C. sinensis* infection prevalence at the country level, data were imported into ArcGIS Pro 2.8.0 (ESRI, Redlands, CA, US) to generate maps.

### Publication bias

Funnel plot analysis was employed to visually assess symmetry in the funnel plots among patients with *O. viverrini* and *C. sinensis* infection. Asymmetry was rigorously evaluated using Egger's approach, where a *P*-value < 0.05 indicated significant publication bias [18].

## Results

### Quality assessment

In assessing the risk of bias in studies related to *O. viverrini* infections, the appraisal revealed that the majority of studies (93.60%, *n* = 117) exhibited low or moderate risk of bias (Figure S1, Tables S2 in Additional file 1). Likewise, in the study of *C. sinensis* infection, most studies (96.15%, *n* = 25) demonstrated low or moderate risk of bias (Figure S2, Table S3 in Additional file 1). These findings underscore the overall standardized methodological quality of the studies included in the systematic review.

### Publication bias

Upon visual inspection of the funnel plot for the prevalence estimate of *O. viverrini* infection, an asymmetry is evident, suggesting a potential publication bias. It appears that smaller studies, potentially yielding more positive outcomes, are published more frequently, as observed with a cluster of studies in the upper left of the plot (see Additional file 1: Figure S3). To validate this visual assessment, we conducted an Egger's test, providing a statistical measure of the funnel plot's asymmetry. The Egger’s test revealed significant small-study effects (*P*-value = 0.001), indicating the presence of publication bias.

Similarly, the funnel plot for the prevalence estimate of *C. sinensis* infection also displayed potential asymmetry, indicating the presence of publication bias. This was particularly noted by the scarcity of studies with high standard errors and low proportion estimates in the upper left of the plot (see Additional file 1: Figure S4). To quantify this potential bias, we performed an Egger’s test, which confirmed statistical significance (*P-value* = 0.006). These observations collectively suggest that publication bias may have influenced the results, potentially leading to an underestimation of the prevalence estimates for *O. viverrini* and *C. sinensis* infections in the GMS.

### Search results

**Fig. 1:**
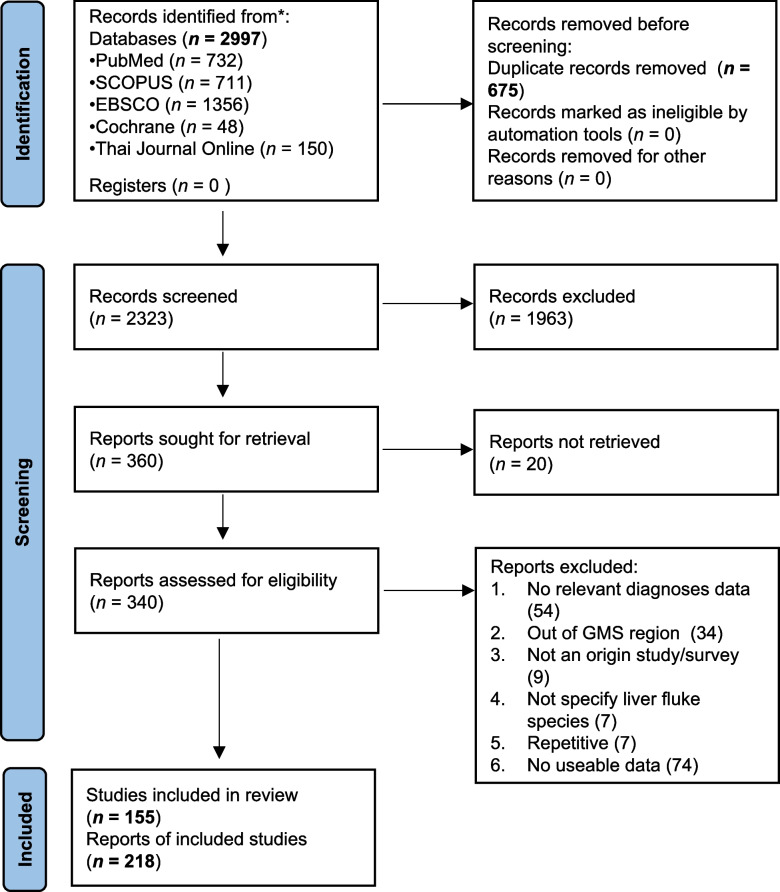
PRISMA flowchart of the available published documents for the prevalence of *Opisthorchis viverrini* and *Clonorchis sinensis* in the GMS

Our electronic database search yielded a total of 2997 articles. After removing 675 duplicates, we screened 2323 studies based on titles and abstracts. Subsequently, 340 studies were retained for further review, involving the removal of duplicate articles and a critical appraisal of titles and abstracts. Following the application of eligibility criteria, 155 articles comprising 218 datasets were deemed suitable for inclusion in the quantitative synthesis (Fig. 1).

### Study characteristics

The datasets encompassed 751,108 participants from six countries, including Thailand, Lao PDR, Cambodia, Vietnam, Myanmar, and China (Guangxi) (Table 1). *Opisthorchis viverrini* datasets were derived from 186 studies (Additional file 2), while *C. sinensis* datasets were sourced from 32 studies (Additional file 3). Studies on *O. viverrini* were conducted primarily in Thailand (89 studies, 47.85%), Lao PDR (55 studies, 29.57%), Cambodia (34 studies, 18.28%), Vietnam (6 studies, 3.23%), and Myanmar (2 studies, 1.07%). The majority of *C. sinensis* studies (24 studies, 75%) were carried out in Guangxi, China, with Vietnam hosting the remaining 25% (8 studies).

**Table 1:** The prevalence of *Opisthorchis viverrini* and *Clonorchis sinensis* in the Greater Mekong subregion

Country (number of datasets available for a particular country)	Number of people screened (total)	Number of test positive people	Pooled prevalence, % (95% *CI*)	*I* ^*2*^
***Opisthorchis viverrini*** ** (186)**	**260,237**	**45,083**	**21.11 (17.74–24.47)**	**99.8%**
Thailand (89)	169,577	29,182	18.19 (13.86–22.51)	99.9%
Laos (55)	54,457	12,870	34.06 (26.85–41.26)	99.9%
Cambodia (34)	24,927	2468	10.48 (5.52–15.45)	99.1%
Vietnam (6)	8855	531	11.75 (1.25–22.24)	99.2%
Myanmar (2)	2421	32	2.66 (0.00–6.83)	92.7%
***Clonorchis sinensis (32)***	**490,871**	**53,128**	**25.33 (18.32–32.34)**	**99.8%**
China-Guangxi (24)	484,578	52,092	26.89 (18.34–35.43)	99.8%
Vietnam (8)	6293	1036	20.30** (**9.13–31.47)	99.1%

### Prevalence of *Opisthorchis viverrini* and *Clonorchis sinensis* in the GMS

**Fig. 2:**
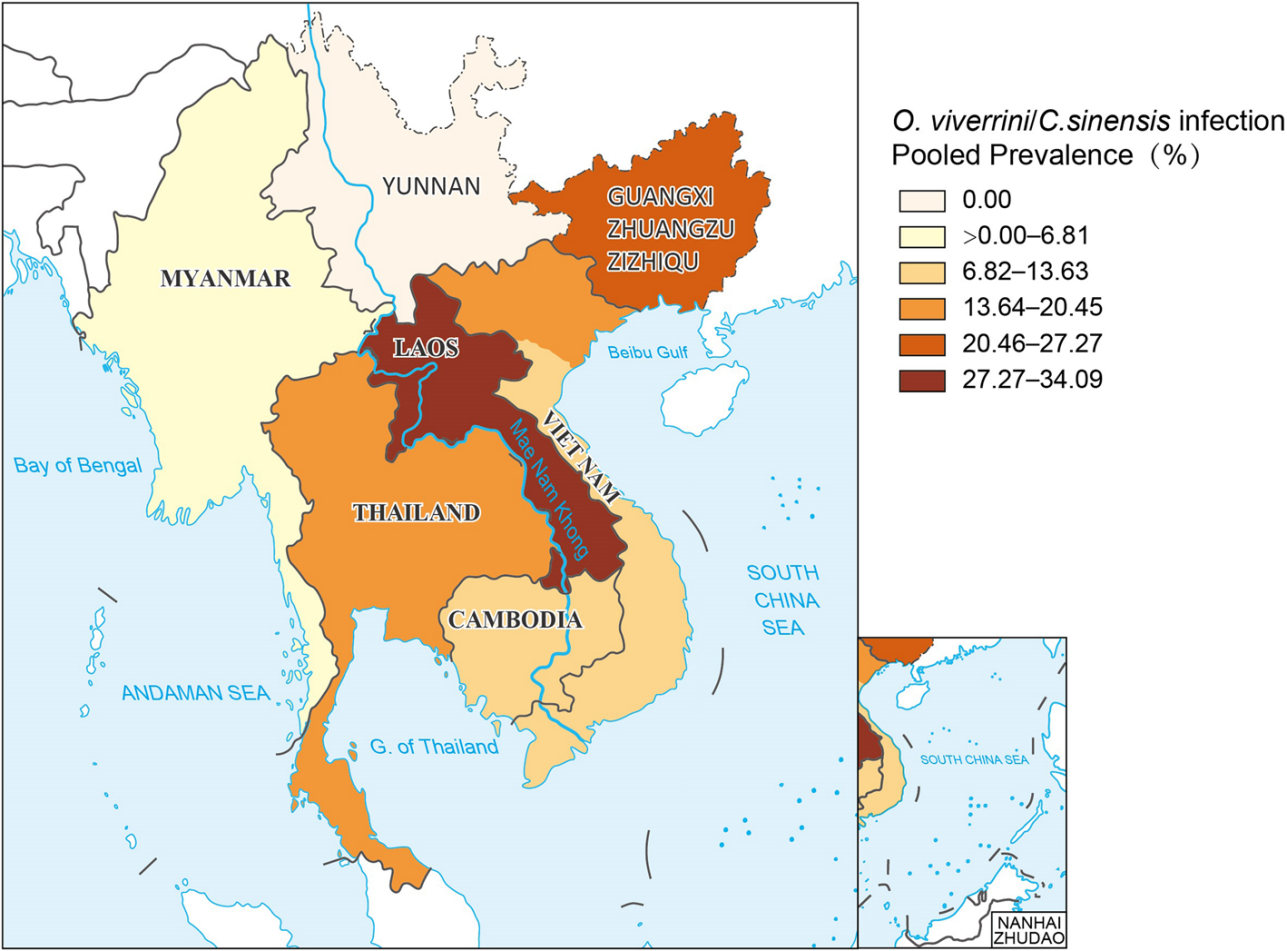
Map depicting the estimated prevalence of *Opisthorchis viverrini* and *Clonorchis sinensis* in the GMS. Map approval No.: GS (2024) 1295

In total, 45,083 individuals out of a general population of 260,237 tested positive for *O. viverrini*, yielding a pooled prevalence of 21.11% (95% *CI*: 17.74–24.47%). Notably, there was significant heterogeneity among studies (*I* ^2^ = 99.80%, *P*-value < 0.001). The highest pooled prevalence estimates were observed in Lao PDR (34.06%, 95% *CI*: 26.85–41.26%), Thailand (18.19%, 95% *CI*: 13.86–22.51%), Vietnam (11.75%, 95% *CI*: 1.25–22.24%), and Cambodia (10.48%, 95% *CI*: 5.52–15.45%), while Myanmar exhibited the lowest prevalence at 2.66% (95% *CI*: 0.00–6.83%) (Table 1, Fig. 2).

Regarding *Clonorchis sinensis* infection in the GMS, the overall prevalence is 25.33% (95% *CI*: 18.32–32.34%). Guangxi, China, recorded the highest prevalence rates at 26.89% (95% *CI*: 18.34–35.43%), whereas Vietnam had lower prevalence rates at 20.30% (95% *CI*: 9.13–31.47%) (Table 1, Fig. 2).

**Fig. 3:**
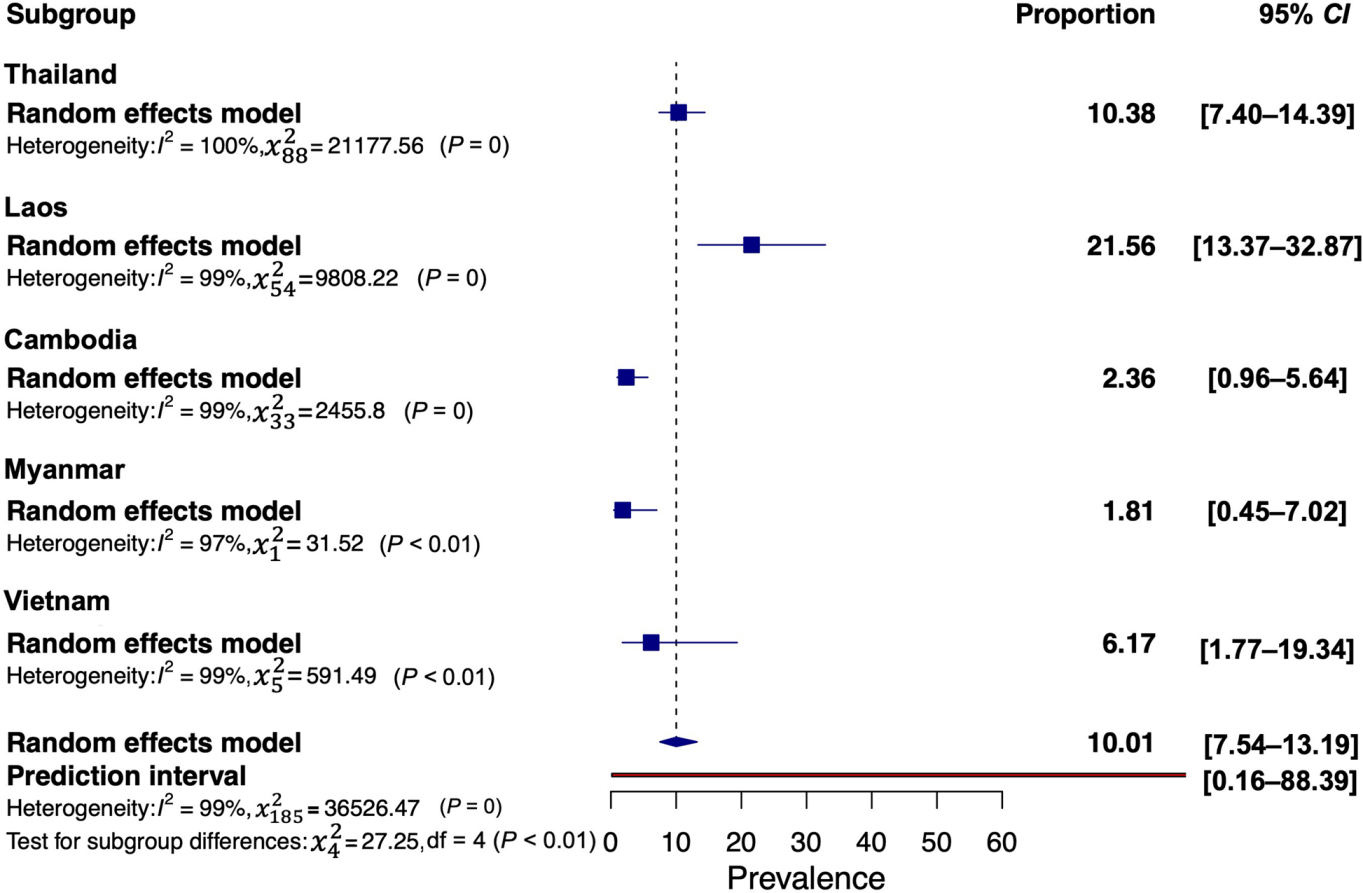
Forest plot of subgroup analysis with country segregation to examine opisthorchiasis in GMS

**Fig. 4:**
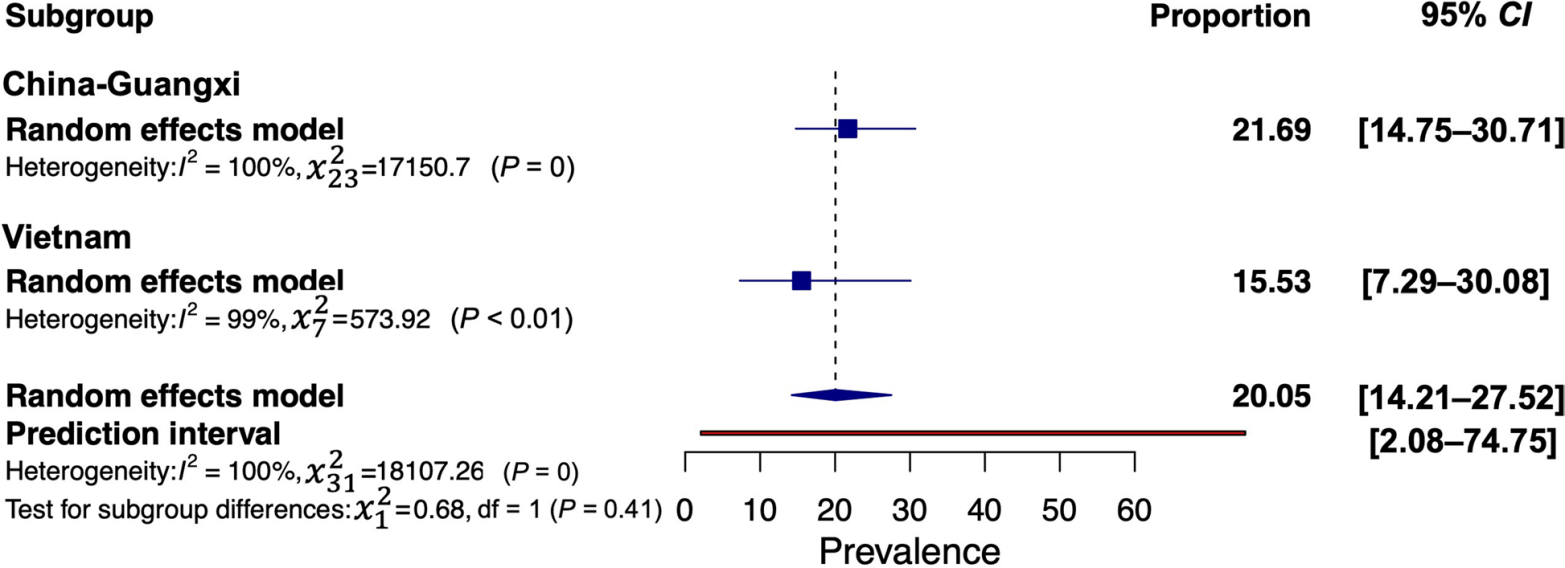
Forest plot of subgroup analysis with country segregation to examine clonorchiasis in GMS

The subgroup analysis, based on different GMS countries, is depicted in Figs. 3 and 4, showing trends similar to the pooled prevalence as presented in Table 1. The prevalence of *O. viverrini* infection was highest in Lao PDR (21.56%, 95% *CI*: 13.37–32.87%), followed by Thailand (10.38%, 95% *CI*: 7.40–14.39%), Vietnam (6.17%, 95% *CI*: 1.77–19.34%), Cambodia (2.36%, 95% *CI*: 0.96–5.64%), and Myanmar (1.81%, 95% *CI*: 0.45–7.02%) (Fig. 3). The subgroup analysis of *C. sinensis* infection revealed prevalence rates ranging between 15.53% and 21.69%. After Guangxi, China (21.69%, 95% *CI*: 14.75–30.71%), Vietnam (15.53%, 95% *CI*: 7.29–30.08%) exhibited the second-highest pooled prevalence (Fig. 4).

### Prevalence of *Opisthorchis viverrini* infection over time in the GMS

According to the study population in the GMS, 35 datasets were conducted in children, 90 datasets were examined in both children and adults across all age groups, and 61 studies were conducted in adults. Subgroup analyses revealed that the *O. viverrini* infection rate was 2.87% (95% *CI*: 1.44–5.64%) among children, 14.84% (95% *CI*: 9.81–21.82%) across all age categories, and 10.98% (95% *CI*: 7.49–15.80%) among adults (Table 2).

**Table 2:** Prevalence estimates of *Opisthorchis viverrini* and *Clonorchis sinensis* infection in the GMS, according to a priori-defined subgroups and age group parameters

Variable: subgroup	*O. viverrini*			*C. sinensis*		
	**Number datasets**	**Pooled prevalence, % (95% ** ***CI*** **)**	***I*** ^**2**^	**Number datasets**	**Pooled prevalence, % (95% ** ***CI*** **)**	***I*** ^**2**^
**Age groups**			99.5%			99.8%
**Children**	35	2.87 (1.44–5.64) *	99.7%	-	-	-
**Adult**	61	10.98 (7.49–15.80)	98.6%	4	13.09 (3.78–36.58)	98.1%
**All ages**	90	14.84 (9.81–21.82)	99.2%	28	21.13 (14.81–29.24)	99.8%
**Collectionperiod**			99.5%			99.8%
**Before 2000**	30	29.27 (16.94–45.63) *	99.6%	5	20.85 (8.19–43.77)	99.1%
**2000–2009**	49	4.32 (2.10–8.70)	99.3%	9	13.00 (6.45–24.48)	99.6%
**2010–2019**	103	10.69 (7.72–14.64)	99.4%	17	25.58(16.90–36.73)	99.8%
**2020-present**	4	1.86 (0.62–5.48)	83.8%	-	-	-

^*^Subgroup was considered as statistically significant if the *P* value was < 0.0.5

**Fig. 5:**
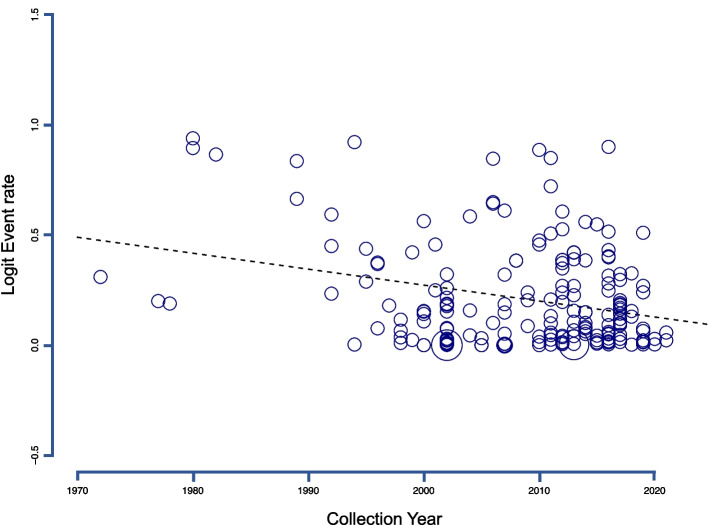
Random-effects meta-regression analyses illustrating the prevalence trend of *Opisthorchis viverrini* infection in the general population across different collection periods. The results reveal a statistically significant downward trend in prevalence within the GMS

An additional subgroup analysis was performed to examine the temporal pattern of *O. viverrini* infection, focusing on the timeframe of sample collection. Based on the period of collecting the samples, the subgroup analysis showed that the overall prevalence rates of *O. viverrini* infection in the GMS were 29.27% (95% *CI*: 16.94–45.63%), 4.32% (95% *CI*: 2.10–8.70%), 10.69% (95% *CI*: 7.72–14.64%), and 1.86% (95% *CI*: 0.62–5.48%) for studies released before the year 2000, between 2000 and 2009, between 2010 and 2019, and from 2020 to the present, respectively (Table 2). Random-effects meta-regression analysis revealed a significant substantial declining trend in prevalence with the collection period (*P*-value < 0.0001) (Fig. 5). It's important to note that Vietnam and Myanmar had limited access data for such analysis (6 for Vietnam and 2 for Myanmar), and therefore, subgroup analysis and meta-regression were omitted to avoid excessive estimated errors.

### Prevalence of *Opisthorchis viverrini* Infection in different diagnostic methods

Various diagnostic techniques were employed for opisthorchiasis diagnosis in the datasets, categorized into three main groups: 1) the Kato-Katz method (90 datasets), 2) FECT (49 datasets), and 3) other techniques, including sedimentation, flotation methods, Stoll's dilution egg count, and/or molecular techniques (44 datasets). These diagnostic methods were further divided into four collecting periods to illustrate the progression of diagnostic techniques over time, with prevalence estimates of *O. viverrini* infection in the GMS shown in Table 3.

**Table 3:** Prevalence estimates of *Opisthorchis viverrini* infection in the GMS, as determined by methods of diagnosis over time

**Variable: subgroup**	***O. viverrini***		
	**Number datasets**	**Pooled prevalence, % (95% ** ***CI*** **)**	***I*** ^***2***^
**Diagnostic method used with 4 collection periods**			
**Before 2000**			99.9%
Kato Katz	8	32.83 (12.23–63.15)	99.5%
FECT	7	20.45 (8.41–41.84)	98.6%
Others	15	32.37 (13.45–59.59)	99.7%
**2000–2009**			99.7%
Kato Katz	23	4.98(4.88–5.03)	99.3%
FECT	4	6.31 (1.85–19.40)	99.4%
Others	22	3.28 (1.37–7.66)	98.7%
2010–2019*			99.7%
Kato Katz	59	15.73 (10.49–22.92) *	99.5%
FECT	34	8.20 (4.90–13.39)	99.3%
FECT and/or Kato-Katz	3	2.58 (0.42–14.31)	97.6%
Others	7	2.23 (0.66–7.29)	97.2%
**2020–present**			87.9%
FECT	4	2.58 (0.09–5.07)	87.9%

^*^Subgroup was considered as statistically significant if the *P-*value was < 0.0.5

In each period of data collection, subgroup analysis revealed that the prevalence rates of diagnostic methods before the year 2000 and between 2000 and 2009 did not differ significantly (*P*-value = 0.65, 0.59, respectively). However, a significant difference (*P*-value = 0.0029) was observed among diagnostic methods during 2010–2019. Specifically, subgroup analysis of diagnostic methods during 2010–2019 revealed pooled prevalence rates of 15.73% (95% *CI*: 10.49–22.92%) for Kato-Katz, 8.20% (95% *CI*: 4.90–13.39%) for FECT, 2.58% (95% *CI*: 0.42–14.31%) for a combination of FECT and Kato-Katz, and 2.23% (95% *CI*: 0.66–7.29%) for other diagnostic methods, respectively.

### Prevalence of *Clonorchis sinensis* infection over time in the GMS

**Fig. 6:**
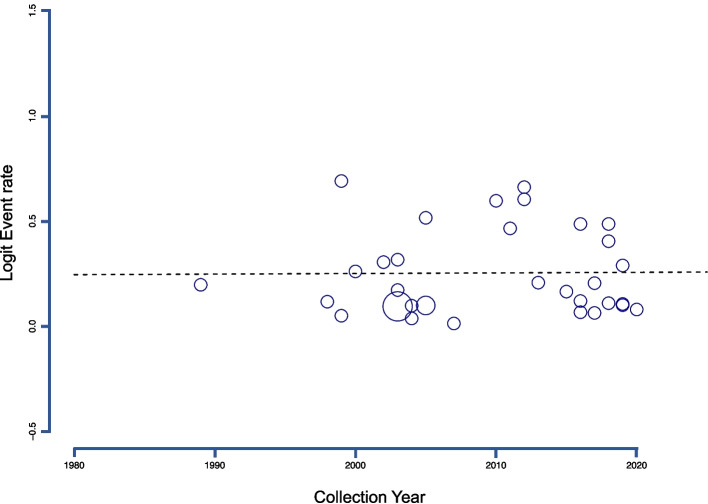
Random-effects meta-regression analysis of *Clonorchis sinensis* infection prevalence in the general population across collection periods showed a statistically non-significant constant trend in the GMS

Subgroup analysis of *C. sinensis* infection in the GMS, specifically in Vietnam and China (Guangxi), was conducted based on age groups and data collection periods. For age group analysis, the pooled prevalence was found to be 13.09% (95% *CI*: 3.78–36.58%) in adults and 21.13% (95% *CI*: 14.81–29.24%) for all age groups. Regarding the collection periods, the subgroup analysis revealed prevalence rates of 20.85% (95% *CI*: 8.19–43.77%), 13.00% (95% *CI*: 6.45–24.48%), and 25.58% (95% *CI*: 16.90–36.73%) for studies conducted before the year 2000, between 2000 and 2009, and from 2010 to 2019, respectively (Table 2). However, the differences between collection periods were not statistically significant (*P*-value = 0.08). A random-effects meta-regression analysis further revealed a non-significant constant trend in the prevalence rates across collection periods (*P*-value = 0.93) (Fig. 6).

### Prevalence of *Opisthorchis viverrini* infection in Thailand

In Thailand, 89 out of 186 research studies reported the prevalence of *O. viverrini* infection, involving a total study population of 169,577 (Table 1). The pooled prevalence of infection among Thais was found to be 18.19% (95% *CI*: 13.86 to 22.59%) (Table 1). Significant heterogeneity between studies was observed, with population type, collection period, and region serving as significant covariates.

The infection prevalence was substantially greater across all age groups at 14.84% (95% *CI*: 8.63–24.32%) compared to adults (9.29%, 95% *CI*: 6.32–13.46%) and children (1.22%, 95% *CI*: 0.33–4.43%), indicating a significant difference (*P*-value = 0.002). The trend of *O. viverrini* infection in Thailand showed a decline over time, being significantly higher before the year 2000 at 40.86% (95% *CI*: 21.50–63.54%) compared to 1.13% (95% *CI*: 0.37–3.37%) two decades later from 2020 to the present. Furthermore, over the more than two decades of data collection in Thailand, random-effects meta-regression analysis revealed a significant downward trend in prevalence (*P*-value = 0.0001) (Additional file 1: Figure S5).

In regions where sufficient data allowed for the evaluation of study region as a covariate, the *O. viverrini* infection prevalence was significantly higher in the Northeast (13.66%, 95% *CI*: 9.44–19.36%) and the North (5.53%, 95% *CI*: 2.57–11.51%, *P*-value = 0.007) compared to other regions of Thailand (Table 4).

**Table 4:** Prevalence estimates of *Opisthorchis viverrini* infection in Thailand, Lao PDR, and Cambodia, according to a priori-defined subgroups and age group parameters

Variable: subgroup	Thailand			Lao PDR			Cambodia		
	Number datasets	Pooled prevalence, % (95% *CI*)	*I* ^*2*^	Number datasets	Pooled prevalence, % (95% *CI*)	*I* ^*2*^	Number datasets	Pooled prevalence, % (95% *CI*)	*I* ^*2*^
**Age groups**									
Children	5	1.22 (0.33–4.43) *	95.2%	22	4.66 (2.04–10.29) *	98.9%	8	1.05 (0.18–5.88)	97.2%
Adult	44	9.29 (6.32–13.46)	99.3%	8	40.47 (19.14–66.17)	99.1%	9	5.58 (1.29–21.11)	97.4%
All ages	40	14.84 (8.63–24.32)	99.7%	25	48.24 (35.28–61.44)	99.0%	17	2.29 (0.63–8.02)	99.1%
**Collection period**									
Before 2000	16	40.86 (21.50–63.54) *	99.8%	6	54.94 (41.00–68.17) *	95.4%	4	5.14 (2.16–11.78) *	98.5%
2000–2009	10	13.97 (9.54–20.00)	99.0%	26	7.96 (3.35 –17.75)	99.5%	17	0.17 (0.01–2.72)	98.6%
2010–2019	60	7.08 (4.92–10.08)	99.3%	23	40.18 (26.41–55.71)	99.0%	13	6.19 (2.65–13.76)	97.2%
2020-present	3	1.13 (0.37–3.37)	67.8%	-	-	-	-	-	-
**Region (Thailand)**									
Northeast	65	13.66 (9.44–19.36) *	99.5%						
North	13	5.53 (2.57–11.51)	98.9%						
Others	8	3.69 (0.76–16.07)	99.1%						
countrywide	3	4.64 (2.42–8.69)	99.7%						
**Provinces (Lao PDR)**									
Saravane				5	62.53 (35.79–83.32) *	99.6%			
Khammouane				6	46.82 (38.18–55.65)	97.4%			
Champasack				13	45.52 (24.42–68.36)	99.3%			
Savannakhet				4	30.38 (23.7–37.90)	84.7%			
Vientiane				9	30.54 (14.87–52.53)	99.0%			
Luang Prabang				3	8.30 (2.62–23.35)	98.3%			
Mixed				4	12.93 (2.31–48.28)	99.7%			
Others				11	1.53 (0.43–5.23)	98.2%			
**Provinces (Cambodia)**									
Kampong Cham							3	26.95 (16.42–40.94) *	96.1%
Kratie							6	2.96 (0.71–11.46)	98.7%
Takeo							5	0.69 (0.03–15.44)	98.1%
Mixed							3	7.61 (2.17–23.42)	98.0%
Others							17	1.29 (0.29–5.48)	97.6%

The blank means those countries were not fit to specific subgroup which is uniqe in each country such as province name

^*^Subgroup was considered as statistically significant if the *P-*value was < 0.05. -: Not applicable

### Prevalence of *Opisthorchis viverrini* Infection in Lao PDR

In Lao PDR, 55 studies involving a total of 54,457 participants reported a pooled *O. viverrini* infection prevalence of 34.06% (95% *CI*: 26.85–41.26%) (Table 1). Table 4 displays the prevalence of infection with significantly high heterogeneity (*I* ^*2*^ = 99.9%, *P*-value < 0.0001). Population type, collection period, and province served as significant variables contributing to the significant heterogeneity among studies.

High infection prevalence was observed across all age groups, at 48.24% (95% *CI*: 35.28–61.44%), compared to adults (40.47%, 95% *CI*: 19.14–66.17%) and children (4.66%, 95% *CI*: 2.04–10.29%) (Table 4, *P*-value < 0.0001). Before the year 2000 through 2019 in Lao PDR, a random-effects meta-regression revealed a non-significant upward trend in prevalence (*P*-value = 0.27) (Additional file 1: Figure S6). Among the periods of data collection, opisthorchiasis prevalence was significantly higher before the year 2000 at 54.94% (95% *CI*: 41.00–68.17%) and remained high at 40.18% (95% *CI*: 26.41–55.71%) in the later two decades, except for the period between 2000 and 2009 when the prevalence decreased to 7.96% (95% *CI*: 3.35–17.75%). Only one study on *O. viverrini* prevalence from 2020 to the present was available, and it remained high at 51.04% (95% *CI*: 48.04–54.05%).

For the endemic locations with provided data, three provinces exhibited considerably higher prevalence than others, namely, Saravane (62.53%, 95% *CI*: 35.79–83.32%), Khammouane (46.82%, 95% *CI*: 38.18–55.65%), and Champasack (45.52%, 95% *CI*: 24.42–68.36%) (Table 4).

### Prevalence of *Opisthorchis viverrini* Infection in Cambodia

Out of 186 articles published on *O. viverrini* infection prevalence, a total of 34, with a population of 24,927, were recruited from Cambodia. The prevalence of *O. viverrini* infection in Cambodia was 10.48% (95% *CI*: 5.52–15.45%) (Table 1). Significant variation in prevalence was observed among studies, including collecting period and province, but not among age groups (Table 4). Prevalence was slightly higher among adults (5.58%, 95% *CI*: 1.29–21.11%) compared to all ages (2.29%, 95% *CI*: 0.63–8.02%) and children (1.05%, 95% *CI*: 0.18–5.88%), but the difference was not statistically significant (*P*-value = 0.60) (Table 4).

Regarding the time period, opisthorchiasis prevalence was reported to be low prior to the year 2000 at 5.14% (95% *CI*: 2.16–11.78%) and slightly increased to 6.19% (95% *CI*: 2.65–13.76%) between 2010 and 2019, with a substantial reduction to 0.17% (95% *CI*: 0.01–2.72%) between 2000 and 2009. Random-effects meta-regression analysis revealed a non-significant increasing trend in the collecting period of prevalence rates (*P*-value = 0.12) (Additional file 1: Figure S7). Although inadequate data were available to explore all provinces, one province named Kampong Cham had the highest prevalence (26.95%, 95% *CI*: 16.42–40.94%), while Kratie and Takeo had the lowest (2.96%, 95% *CI*: 0.71–11.46% and 0.69%, 95% *CI*: 0.03–15.44%, respectively) (Table 4).

### Prevalence of *Clonorchis sinensis* Infection in Guangxi, China

Using random-effects analysis, the pooled prevalence estimate of *C. sinensis* infection in Guangxi, China, was determined to be 26.89% (95% *CI*: 18.34–35.43%) with considerable heterogeneity (*I* ^*2*^ = 99.8%, *P*-value = 0.001). The study by Hong et al. (2001) identified the highest proportion of *C. sinensis* infection (69.00%, 95% *CI*: 58.97–77.87%) [19]. Since the demographic datasets did not include age information, subgroup analysis based on age was not performed.

For the time period subgroup analysis, prevalence rates of 42.10% (95% *CI*: 13.55–77.13%), 12.72% (95% *CI*: 6.75–22.69%), and 25.42% (95% *CI*: 15.99–37.90%) were observed for data collected before the year 2000, between 2000 and 2009, and from 2010 to 2019, respectively (Table 5). There was no significant difference among the periods of data collection. However, a slightly decreasing trend in the prevalence rates of *C. sinensis* over the collection periods was observed through random-effects meta-regression analysis (*P*-value = 0.46) (Additional file 1: Figure S8).

**Table 5:** Prevalence estimates of *Clonorchis sinensis* infection in Guangxi, China, and Vietnam, according to a priori-defined subgroups and demographic parameters

Variable: subgroup	**Guangxi, China**			**Vietnam**		
	Number datasets	Pooled prevalence, % (95% *CI*)	*I* ^2^	Number datasets	Pooled prevalence, % (95% *CI*)	*I* ^2^
**Age groups**						
Children	-	-	-	-	-	-
Adult	-	-	-	4	13.09 (3.78–36.58)	98.1%
All ages	-	-	-	4	17.88 (6.89–39.05)	99.0%
**Collection period**						
Before 2000	2	42.10 (13.55–77.13)	99.0%	3	11.85 (5.23–24.66)	99.3%
2000–2009	6	12.72 (6.75–22.69)	99.8%	3	12.82 (1.85–53.35)	94.5%
2010–2019	15	25.42 (15.99–37.90)	99.9%	2	26.88 (13.47–46.47)	98.7%
2020-present	-	-	-	-	-	-

^*^Subgroup was considered as statistically significant if the *P-*value was < 0.05. -: Not applicable

### Prevalence of *Clonorchis sinensis* infection in Vietnam

In Vietnam, a total of 1036 individuals from a population of 6293 tested positive for *C. sinensis* infection, indicating a pooled prevalence of 20.30% (95% *CI*: 9.13–31.47%). Subgroup analysis based on the collection time revealed prevalence rates of 11.85% (95% *CI*: 5.23–24.66%), 12.82% (95% *CI*: 1.85–53.35%), and 26.88% (95% *CI*: 13.47–46.47%) for studies collected before 2000, between 2000 and 2009, and from 2010 to 2019, respectively (Table 5). A non-significant, upward trend in prevalence rates over time was detected using random-effects meta-regression analysis (*P*-value = 0.31) (Additional file 1: Figure S9).

While the difference between demographic groups was not statistically significant (*P*-value = 0.68), it was observed to be higher among all ages (17.88%, 95% *CI*: 6.89–39.05%) than among adults (13.09%, 95% *CI*: 3.78–36.58%) (Table 5).

## Discussion

Opisthorchiasis and clonorchiasis are major neglected tropical diseases associated with poverty, representing significant yet overlooked foodborne trematodiases in the GMS [2]. These liver fluke diseases not only qualify as helminthic infectious diseases but also as carcinogenic parasites, posing a substantial risk of cancer with elevated mortality rates in this region [2, 20, 21]. Khon Kaen Province in Northeast Thailand has reported the highest incidence of cholangiocarcinoma in the world [3, 5]. Individuals afflicted with liver fluke diseases not only face health challenges but also bear the brunt of socioeconomic losses that exacerbate their existing poverty [7]. However, the evolving burden of these infections in the GMS remains unknown. In this study, we present, for the first time, a systematic review and meta-analysis aiming to elucidate the pooled prevalence estimates and time trends of *O. viverrini* and *C. sinensis* infections in the GMS.

Opisthorchiasis, attributed to *O. viverrini*, has been recognized as endemic in Thailand for over a century [22, 23]. Subsequently, there have been substantial reports of opisthorchiasis in the neighboring Mekong countries. This systematic review includes information from at least 186 eligible original articles, providing insights into the prevalence of *O. viverrini* infection in the GMS, specifically Thailand, Lao PDR, Cambodia, Vietnam, and Myanmar. The overall pooled prevalence of *O. viverrini* infection in the GMS is considerable, with a rate of 21.1%, though its distribution among the countries is uneven. Lao PDR exhibited the highest prevalence (34.1%), followed by Thailand (18.2%), Vietnam (11.8%), Cambodia (10.5%), and Myanmar (2.7%). The latter three countries showed high heterogeneity in prevalence, as indicated by the marked 95% confidence interval, similar to clonorchiasis in Guangxi, China, and Vietnam (Table 1). The observed differences in prevalence may be attributed to several factors, including insufficient survey coverage and/or the use of different diagnostic tests [24]. Low screening coverage, both geographically and in the number of cases examined, can be found in datasets from Vietnam, Myanmar, and Cambodia. The asymmetry of the funnel plots for both opisthorchiasis and clonorchiasis in our study clearly supports this conclusion.

For the diagnostic methods of opisthorchiasis and clonorchiasis, Kato-Katz was commonly employed, as indicated by the datasets presented in this systematic review (Table 3). Overall, different diagnostic methods showed no obvious effect on the pooled prevalence. The diagnostic sensitivity of Kato-Katz is generally lower than that of FECT, particularly in cases of light infections [25–27]. However, comparable or even higher sensitivity has been reported [28–30]. The higher prevalence of opisthorchiasis with Kato-Katz over other methods (FECT and others) in this review during the years 2010 to 2019 is likely attributed to the datasets recruited from different geographical locations rather than the diagnostic methods. Specifically, Kato-Katz was a commonly used technique in Lao PDR, where over 63.2% (5622 out of 8890) of opisthorchiasis cases were reported. Moreover, most of the cases were from high-endemic provinces, including Saravane, Champasak, and Khammouane (Table 4) [31, 32]. In contrast, FECT, which was mostly employed in Thailand (25 of 34 datasets), reported a prevalence of 7.1% for *O. viverrini* infection during the same period (Table 4). These differences can cause misinterpretation if not considered. However, it's important to note that all reported prevalences of opisthorchiasis or even clonorchiasis by stool examination may be underestimated, given that no eggs were found when the worm burden is less than 20 or approximately 1000 eggs per gram [26, 33].

The prevalence of opisthorchiasis exhibited a distinct time trend, with noticeably higher prevalence evident in datasets collected before the year 2000 compared to those gathered thereafter (Table 2). The significant decline in opisthorchiasis prevalence in Thailand (40.9%, 14%, 7%, and 1% before the year 2000, 2000–2009, 2010–2019, and 2020—present, respectively) (Table 4) may reflect the impact of continuous opisthorchiasis integrated control programs. These programs include nationwide surveillance conducted every 5 years up to the present [2, 34–36]. Moreover, the striking decline in the overall pooled prevalence of opisthorchiasis from 29.3% to 4.3% during the period from 2000 to 2009 (Table 2) may be attributed to the reduction in prevalence observed in Lao PDR and Cambodia (Table 4). This reduction can be linked to a deworming campaign targeting *Schistosoma mekongi*, soil-transmitted helminths (STH), and foodborne trematodiases conducted in the late 1990s to the early 2000s in Lao PDR and Cambodia [37]. Consequently, the prevalence of opisthorchiasis in these two countries was lower than in other periods of data collection. Furthermore, Cambodia implemented mass drug administration (MDA) programs involving praziquantel in combination with mebendazole annually from 2002 to 2004 [38, 39]. Following the conclusion of these campaigns in both Lao PDR and Cambodia, the prevalence of opisthorchiasis rebounded to an even higher rate than observed between the years 2000 and 2009, as seen in Cambodia (Table 4). Therefore, while the overall opisthorchiasis prevalence in the GMS showed a downward trend over time (Fig. 5), there was an upward trend in Lao PDR and Cambodia.

For clonorchiasis, observed only in Guangxi and the northern parts of Vietnam, the prevalence and trend remain consistently high throughout the data collection periods. Similar to opisthorchiasis, the pooled prevalence of clonorchiasis was higher in the years before 2000, but it is not significantly different from the years thereafter (Table 5). Several reports have documented an increase in clonorchiasis prevalence since 1989 in Guangxi, China [40, 41], particularly in Hengxian county, Nanning district [42].Despite the Ministry of Health's 2006–2015 National Plan for the Prevention and Control of Key Parasitic Diseases implementing mass and selective chemotherapy in areas with prevalence rates of over 40% and between 10 and 40%, reinfection continued to occur [43]. In Nanning, Guangxi, the incidence of reinfection can reach 64 per 100 person-years [43]. The main risk factors associated with sustained infection in Guangxi may be males, the Zhuang ethnic minority, and eating behavior [44]. This is evident as a maximum of 60.6% of people reported that they intended to keep eating raw fish despite knowing the risk of infection [45]. The high economic development of China over the past decades has fostered the growth of aquaculture, making it economically feasible for more residents to consume freshwater fish dishes, a source of *C. sinensis* metacercariae, which has become increasingly popular in certain counties of Guangxi [40].

In Vietnam, the pooled prevalence of clonorchiasis increased during the years 2010 to 2019, although it is not significantly different from the earlier periods. This could be attributed to more endemic foci being investigated in the northern parts of the country over the past decades, such as the Thac Ba lake region, Yen Bai, and Thanh Hoa provinces, where the prevalence of *C. sinensis* infection is high (40.4%) with prevalence at the commune level ranging between 26.5% and 53.3% [46] The incidence rate of fishborne trematode reinfection was as high as 21.4/100 person-years [47]. Major risk factors for infection include the behavior of eating raw fish, low education level, lack of treatment, and poor hygienic sanitation [46–48].

Nationwide intervention and mass drug administration are not practiced in both Vietnam and Guangxi, China [40]. Therefore, a stably high prevalence of clonorchiasis persists in these countries. Addressing *C. sinensis* infection in the GMS requires systematic surveillance and intervention with a holistic approach to effectively combat this parasite. Increased awareness campaigns are needed to discourage the consumption of raw fish in these areas and reduce the incidence of liver fluke infection [2, 41, 46, 48]

Based on our study's findings, a significant implication is the critical need for ongoing and enhanced public health interventions in the GMS to manage liver fluke infections. The comprehensive analysis of *O. viverrini* and *C. sinensis* prevalence over time underscores the persistent and substantial burden these infections place on the region. The data provided could be instrumental in guiding targeted strategies for disease control, including improving diagnostic methods, raising awareness of infection risks, and implementing effective treatment programs. These efforts have the potential to reduce infection rates and associated health burdens in the GMS.

The findings of the meta-analysis should be interpreted with caution due to several limitations. A notable point is the observed between-study heterogeneity in the prevalence estimates of *O. viverrini* and *C. sinensis*. This variation was largely attributed to three covariates that assessed in the subgroup analyses: geographical differences across the GMS, which reflect varied environmental and socio-cultural influences on infection rates; differences in diagnostic methods used for detecting these liver flukes, with different techniques having varying sensitivities and specificities; and the age range of participants, as age-specific factors like susceptibility and exposure levels can significantly impact prevalence. These factors collectively suggest that future research should prioritize standardized diagnostic methods, cover a wider geographical scope, and include diverse age groups to enhance the accuracy and generalizability of prevalence estimates for *O. viverrini* and *C. sinensis*.

In addition, the results of our meta-analysis may be influenced by publication bias, potentially leading to an underestimation of the true prevalence estimates for *O. viverrini* and *C. sinensis* infections in the GMS. The asymmetrical distribution observed in the funnel plots underscores the need for caution when interpreting these findings and indicates that more comprehensive research is necessary to accurately assess the prevalence of both liver flukes in the GMS.

## Conclusions

Opisthorchiasis and clonorchiasis stand out as major neglected foodborne trematodiases in the GMS. Our systematic review involved a comprehensive search for published articles, encompassing over 218 datasets and results for 751,108 people in the GMS over the past decades. This represents one of the most thorough assessments of the epidemiology of *O. viverrini* and *C. sinensis*. The prevalence of *O. viverrini* in the GMS was notably high in Lao PDR, followed by Thailand and Cambodia. Limited data sources on opisthorchiasis in Myanmar and central and southern Vietnam affected the precision of prevalence calculations. *C. sinensis* infection was prominent in Guangxi, China, and the northern parts of Vietnam. Over time, *O. viverrini* prevalence showed a significant decrease in the GMS, while *C. sinensis* infection appeared to remain consistently stable in both China and Vietnam. This study provides an in-depth, systemic prevalence review of human liver flukes in the GMS, highlighting the imperative need for systematic surveillance, data collection, and targeted interventions in the GMS, particularly focusing on regions like Cambodia, Myanmar, Vietnam, and Guangxi in China. Strengthening these efforts is crucial for improving human health and combating these persistent infections in the region.

### Supplementary Information

**Supplementary Material 1.**

**Supplementary Material 2.**

**Supplementary Material 3.**

## Data Availability

All data generated or analyzed during this study are included in this published article and its supplementary information files.

## Abbreviations

the GMS: The Great Mekong Subregion
CCA: Cholangiocarcinoma
NTDs: Neglected tropical diseases
DALYs: Disability-adjusted life years
Lao PDR: Lao People's Democratic Republic
PRISMA: Preferred Reporting Items for Systematic Reviews and Meta-Analyses
JBI: Joanna Briggs Institute
FECT: Formalin-ether concentration technique
95% *CI*: 95% Confidence interval

## References

[1] Qian MB, Zhou XN (2021). Clonorchis sinensis. Trends Parasitol.

[2] Sripa B, Suwannatrai AT, Sayasone S, Do DT, Khieu V, Yang Y (2021). Current status of human liver fluke infections in the Greater Mekong Subregion. Acta Trop..

[3] Sripa B, Kaewkes S, Sithithaworn P, Mairiang E, Laha T, Smout M (2007). Liver fluke induces cholangiocarcinoma. PLoS Med.

[4] Bouvard V, Baan R, Straif K, Grosse Y, Secretan B, El GF (2009). A review of human carcinogens—Part B: biological agents. Lancet Oncol.

[5] Vatanasapt V, Tangvoraphonkchai V, Titapant V, Pipitgool V, Viriyapap D, Sriamporn S (1990). A high incidence of liver cancer in Khon Kaen Province, Thailand - PubMed. Southeast Asian J Trop Med Public Health.

[6] Phoncharoen R, Sripa B, Bundhamcharoen K (2018). The Burden of Cholangiocarcinoma Attributable to Liver Fluke Infection in Thailand. J Health Syst Res.

[7] Khuntikeo N, Thinkhamrop B, Bundhamcharoen K, Andrews RH, Grundy-Warr C, Yongvanit P (2018). The socioeconomic burden of cholangiocarcinoma associated with *Opisthorchis*
*viverrini* Sensu Lato Infection in Northeast Thailand: a preliminary analysis. Adv Parasitol.

[8] Page MJ, McKenzie JE, Bossuyt PM, Boutron I, Hoffmann TC, Mulrow CD, The PRISMA (2020). statement: an updated guideline for reporting systematic reviews. BMJ.

[9] ADB. Greater mekong subregion economic cooperation program. Asian Development Bank. 2015. https://www.adb.org/sites/default/files/publication/29387/gms-ecp-overview-2015.pdf. Accessed 20 Dec 2023.

[10] Munn Z, MClinSc SM, Lisy K, Riitano D, Tufanaru C (2015). Methodological guidance for systematic reviews of observational epidemiological studies reporting prevalence and cumulative incidence data. Int J Evid Based Healthc.

[11] Zhou S, Shen C (2022). Avoiding definitive conclusions in meta-analysis of heterogeneous studies with small sample sizes. JAMA Otolaryngol Head Neck Surg.

[12] Ouzzani M, Hammady H, Fedorowicz Z, Elmagarmid A (2016). Rayyan-a web and mobile app for systematic reviews. Syst Rev.

[13] Balduzzi S, Rücker G, Schwarzer G (2019). How to perform a meta-analysis with R: a practical tutorial. BMJ Ment Health.

[14] Lipsey M, Wilson D. Practical meta-analysis – IDoStatistics. SAGE Publications. 2001. https://idostatistics.com/lipsey-wilson-2001-practical-meta-analysis-2001/. Accessed 13 Jul 2023.

[15] DerSimonian R, Laird N (1986). Meta-analysis in clinical trials. Control Clin Trials.

[16] Cochran WG (1954). The combination of estimates from different experiments. Biometrics.

[17] Higgins JPT, Thompson SG (2002). Quantifying heterogeneity in a meta-analysis. Stat Med.

[18] Ioannidis JPA, Stanley TD, Doucouliagos H (2017). The power of bias in economics research. Econ Econ Model Constr.

[19] Hong ST, Rim HJ, Min DY, Li X, Xu J, Feng Z (2001). Control of clonorchiasis by repeated treatments with praziquantel. Korean J Parasitol.

[20] Sripa B, Kaewkes S, Intapan PM,  Maleewong W, Brindley PJ (2010). Food-Borne Trematodiases in Southeast Asia.

[21] Valle JW, Kelley RK, Nervi B, Oh DY, Zhu AX (2021). Biliary tract cancer. Lancet.

[22] Leiper RT (1915). Notes of the occurrence of parasites presumably rare in man. J R Army Med Corps.

[23] Sripa B, Nawa Y, Sithithaworn P, Andrews R, Brindley PJ (2012). Discovery of human opisthorchiasis: a mysterious history. Parasitol Int.

[24] Crellen T, Sithithaworn P, Pitaksakulrat O, Khuntikeo N, Medley GF, Hollingsworth TD (2021). Towards evidence-based control of *Opisthorchis**viverrini*. Trends Parasitol.

[25] Charoensuk L, Subrungruang I, Mungthin M, Pinlaor S, Suwannahitatorn P (2019). Comparison of stool examination techniques to detect *Opisthorchis*
*viverrini* in low intensity infection. Acta Trop.

[26] Johansen MV, Lier T, Sithithaworn P (2015). Towards improved diagnosis of neglected zoonotic trematodes using a one health approach. Acta Trop.

[27] Saijuntha W, Duenngai K, Tangkawattana S, Petney TN, Andrews RH, Sithithaworn P (2018). Recent advances in the diagnosis and detection of *Opisthorchis *
*viverrini* sensu lato in human and intermediate hosts for use in control and elimination programs. Adv Parasitol.

[28] Kopolrat KY, Singthong S, Khuntikeo N, Loilome W, Worasith C, Homwong C (2022). Performance of Mini Parasep® SF stool concentrator kit, Kato-Katz, and formalin-ethyl acetate concentration methods for diagnosis of opisthorchiasis in Northeast Thailand. Parasit Vectors.

[29] Lovis L, Mak TK, Phongluxa K, Soukhathammavong P, Sayasone S, Akkhavong K (2009). PCR diagnosis of *Opisthorchis*
*viverrin*i and *Haplorchis*
*taichui* infections in a lao community in an area of endemicity and comparison of diagnostic methods for parasitological field surveys. J Clin Microbiol.

[30] Qian MB, Yap P, Yang YC, Liang H, Jiang ZH, Li W (2013). Accuracy of the Kato-Katz method and formalin-ether concentration technique for the diagnosis of *Clonorchis sinensis*, and implication for assessing drug efficacy. Parasit Vectors.

[31] Phongluxa K, Xayaseng V, Vonghachack Y, Akkhavong K, van Eeuwijk P, Odermatt P (2013). Helminth infection in southern Laos: high prevalence and low awareness. Parasit Vectors.

[32] Sayasone S, Odermatt P, Phoumindr N, Vongsaravane X, Sensombath V, Phetsouvanh R (2007). Epidemiology of *Opisthorchis viverrini* in a rural district of southern Lao PDR. Trans R Soc Trop Med Hyg.

[33] Sithithaworn P, Tesana S, Pipitgool V, Kaewkes S, Thaiklar K, Pairojkul C (1991). Quantitative post-mortem study of *Opisthorchis viverrini* in man in north-east Thailand. Trans R Soc Trop Med Hyg.

[34] Jongsuksuntigul P, Imsomboon T (2003). Opisthorchiasis control in Thailand. Acta Trop.

[35] Wattanawong O, Iamsirithaworn S, Kophachon T, Nak-ai W, Wisetmora A, Wongsaroj T (2021). Current status of helminthiases in Thailand: a cross-sectional, nationwide survey, 2019. Acta Trop.

[36] Wongsaroj T, Nithikathkul C, Rojkitikul W, Nakai W, Royal L, Rammasut P (2014). National survey of helminthiasis in Thailand. Asian Biomedicine.

[37] Montresor A, Cong DT, Sinuon M, Tsuyuoka R, Chanthavisouk C, Strandgaard H (2008). Large-scale preventive chemotherapy for the control of helminth infection in western pacific countries: six years later. PLoS Negl Trop Dis.

[38] Keang H, Odermatt P, Odermatt-Biays S, Cheam S, Degrémont A, Hatz C (2007). Liver morbidity due to *Schistosoma mekongi* in Cambodia after seven rounds of mass drug administration. Trans R Soc Trop Med Hyg.

[39] Sinuon M, Tsuyuoka R, Socheat D, Montresor A, Palmer K (2005). Financial costs of deworming children in all primary schools in Cambodia. Trans R Soc Trop Med Hyg.

[40] Qian MB, Chen YD, Yang YC, Lu MF, Jiang ZH, Wei K (2014). Increasing prevalence and intensity of foodborne clonorchiasis, hengxian county, China, 1989–2011 - volume 20, number 11—November 2014 - emerging infectious diseases journal - CDC. Emerg Infect Dis.

[41] Qian MB, Zhou CH, Zhu HH, Zhu TJ, Huang JL, Chen YD (2020). From awareness to action: NIPD’s engagement in the control of food-borne clonorchiasis. Adv Parasitol.

[42] National Health Commission China. Notice of the ministry of health on printing and distributing the 2006–2015 national key parasitic disease prevention and control plan 2010, updated 2006 Jun 7 [in Chinese]. 2006. http://www.nhc.gov.cn/bgt/pw10604/200606/849930d7814f4e0d83cdbc9ea9de60da.shtml. Accessed 18 Jul 2023.

[43] Li Z, Xin H, Qian MB, Sun J, Yang Y, Chen Y (2022). *Clonorchis**sinensis* reinfection rate and reinfection determinants: a prospective cohort study in Hengxian County, Guangxi, China. J Infect Dis.

[44] Xu M, Jiang Y, Yin J, Cao S, Shen Y, Cao J (2021). Risk Factors for *Clonorchis sinensis* Infection in Residents of Binyang.

[45] Xin H, Yang Y, Jiang Z, Qian M, Chen Y, Li S (2021). An investigation of Human clonorchiasis prevalence in an endemic County in Guangxi Zhuang Autonomous Region, China, 2016. Food Waterborne Parasitol.

[46] Nguyen TTB, Dermauw V, Dahma H, Bui DT, Le TTH, Phi NTT (2020). Prevalence and risk factors associated with *Clonorchis sinensis* infections in rural communities in northern Vietnam. PLoS Negl Trop Dis.

[47] Nguyen TTB, Dermauw V, Bui DT, Dahma H, Le DT, Nguyen HTT (2023). Incidence of fish-borne trematode infections and associated factors: results from a cohort study in highly endemic communities in northern Vietnam. Parasitol Res.

[48] Doanh PN, Nawa Y (2015). *Clonorchis**sinensis* and *Opisthorchis* spp. in Vietnam: Current status and prospects. Trans R Soc Trop Med Hyg..

