# Supplementary material for: Prevalence estimates of Opisthorchis viverrini and Clonorchis sinensis infection in the Greater Mekong subregion: a systematic review and meta-analysis
Source: Infect Dis Poverty. 2024 May 8;13:33. doi: 10.1186/s40249-024-01201-8 (PMC11077858; doi:10.1186/s40249-024-01201-8)
Supplement: Supplementary file 1 — Supplementary Material 1. [file 40249_2024_1201_MOESM1_ESM.docx]

**Supplementary Table S1 – Search strategy**

| Searching |  |
| --- | --- |
| Liver flukes | "opisthorchis viverrini"[All Fields] OR "liver fluke" OR "opisthorchiasis" OR "clonorchis sinensis" OR "clonorchiasis" |
| Prevalence | "prevalence"[Title/Abstract] OR "incidence" OR "Survey" OR "infection rate" OR "percent" OR "percentage" OR "case report" OR "cross sectional" OR "epidemiology" |
| Greater Mekong Subregion | "gms"[Title/Abstract] OR "Greater Mekong subregion" OR "Cambodia" OR "Lao" OR "Lao PDR" OR "Myanmar" OR "Burma" OR "Thailand" OR "Vietnam" OR "China" OR "Yunnan" OR "Guangxi" OR "Guangxi Zhuang” |
| Population | **(human[MeSH Terms]) OR (adult[MeSH Terms]) OR (children[MeSH Terms])) OR ("school children"[All Fields])** |
| Overall | ((("opisthorchis viverrini"[All Fields] OR "liver fluke" OR "opisthorchiasis" OR "clonorchis sinensis" OR "clonorchiasis") ) AND ("prevalence"[Title/Abstract] OR "incidence" OR "Survey" OR "infection rate" OR "percent" OR "percentage" OR "case report" OR "cross sectional" OR "epidemiology")) AND ("gms"[Title/Abstract] OR "Greater Mekong subregion" OR "Cambodia" OR "Lao" OR "Lao PDR" OR "Myanmar" OR "Burma" OR "Thailand" OR "Vietnam" OR "China" OR "Yunnan" OR "Guangxi" OR "Guangxi Zhuang") AND **(human[MeSH Terms]) OR (adult[MeSH Terms]) OR (children[MeSH Terms])) OR ("school children"[All Fields])** |

**Supplementary Figure S1 – Risk of Bias of *Opisthorchis viverrini***


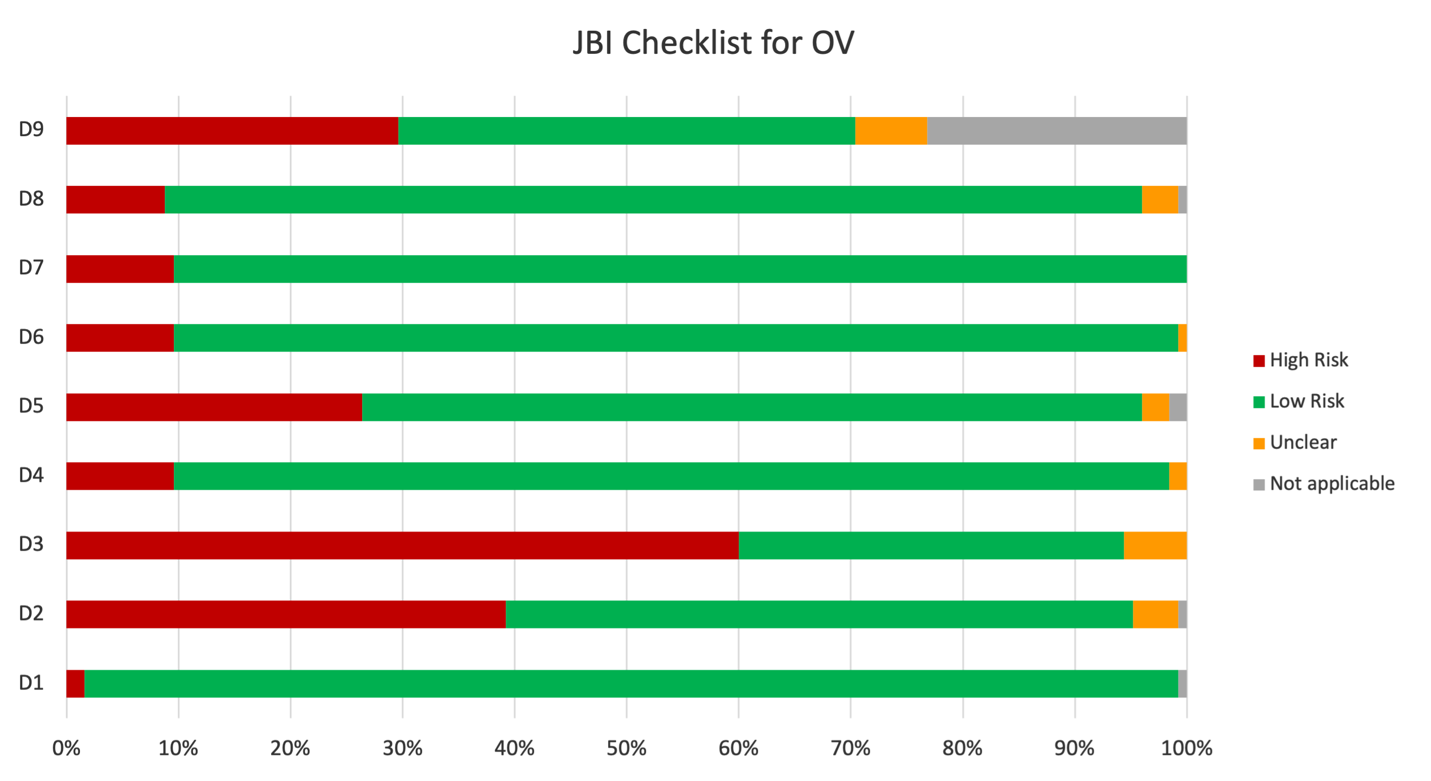


| **Questions:** | |
| --- | --- |
| D1: Was the sample frame appropriate to address the target population? | |
| D2: Were study participants sampled in an appropriate way? | |
| D3: Was the sample size adequate? | |
| D4: Were the study subjects and the setting described in detail? | |
| D5: Was the data analysis conducted with sufficient coverage of the identified sample? | |
| D6: Were valid methods used for the identification of the condition? | |
| D7: Was the condition measured in a standard, reliable way for all participants? | |
| D8: Was there appropriate statistical analysis? | |
| D9: Was the response rate adequate, and if not, was the low response rate managed appropriately? | |
| **Categorical labels:** |  |
| 0= No= High risk, |  |
| 1= Yes= Low risk, |  |
| 2= Un= Unclear |  |
| 3=NA= Not applicable |  |

**Supplementary Table: S2 Risk of bias assessment of included studies for *Opisthorchis viverrini* by JBI critical appraisal checklist for studies reporting prevalence data.**

| Article | First Author, Publication Year | D1 | D2 | D3 | D4 | D5 | D6 | D7 | D8 | D9 | Total | QA grade |
| --- | --- | --- | --- | --- | --- | --- | --- | --- | --- | --- | --- | --- |
| 1 | Sornmani , 1973 | 1 | 0 | 1 | 1 | 1 | 0 | 0 | 1 | 1 | 6 | Medium |
| 2 | Bunnag, 1980 | 1 | 0 | 0 | 1 | 1 | 0 | 0 | 0 | 3 | 3 | High |
| 3 | Sornmani, 1981 | 1 | 1 | 1 | 1 | 1 | 0 | 0 | 1 | 2 | 6 | Medium |
| 4 | Upatham, 1982 | 1 | 0 | 1 | 1 | 1 | 1 | 1 | 1 | 1 | 8 | Low |
| 5 | Upatham, 1984 | 1 | 0 | 1 | 1 | 1 | 0 | 0 | 1 | 3 | 5 | Medium |
| 6 | Upatham, 1985 | 1 | 1 | 1 | 1 | 1 | 0 | 0 | 1 | 1 | 7 | Low |
| 7 | Maleewong, 1992 | 1 | 0 | 1 | 1 | 1 | 1 | 1 | 0 | 0 | 6 | Medium |
| 8 | Radomyos, 1994 | 1 | 1 | 0 | 1 | 1 | 2 | 1 | 1 | 3 | 6 | Medium |
| 9 | Kobayashi, 1996 | 1 | 0 | 0 | 1 | 1 | 1 | 1 | 1 | 1 | 7 | Low |
| 10 | Pungpak, 1997 | 1 | 0 | 0 | 1 | 0 | 0 | 0 | 1 | 1 | 4 | Medium |
| 11 | Vannachone, 1998 | 1 | 0 | 0 | 1 | 0 | 1 | 1 | 1 | 0 | 5 | Medium |
| 12 | Radomyos, 1998 | 1 | 0 | 0 | 0 | 0 | 0 | 0 | 1 | 0 | 2 | High |
| 13 | Kobayashi, 1999 | 1 | 1 | 0 | 2 | 2 | 1 | 1 | 0 | 0 | 4 | Medium |
| 14 | Kobayashi, 2000 | 1 | 0 | 2 | 1 | 1 | 1 | 1 | 1 | 3 | 6 | Medium |
| 15 | Waree, 2001 | 1 | 0 | 0 | 1 | 1 | 1 | 1 | 0 | 3 | 5 | Medium |
| 16 | Sinuon, 2003 | 1 | 0 | 0 | 1 | 1 | 1 | 1 | 1 | 0 | 6 | Medium |
| 17 | Sithithaworn, 2003 | 1 | 0 | 1 | 1 | 1 | 1 | 1 | 0 | 3 | 6 | Medium |
| 18 | Piangjai, 2003 | 1 | 0 | 1 | 1 | 1 | 1 | 1 | 0 | 3 | 6 | Medium |
| 19 | Sriamporn, 2004 | 1 | 0 | 1 | 1 | 1 | 1 | 1 | 1 | 3 | 7 | Low |
| 20 | Sriamporn, 2005 | 1 | 0 | 0 | 1 | 0 | 0 | 0 | 0 | 3 | 2 | High |
| 21 | Nontasut, 2005 | 0 | 0 | 0 | 0 | 0 | 1 | 1 | 1 | 0 | 3 | High |
| 22 | Sithithaworn, 2006 | 1 | 0 | 0 | 1 | 1 | 1 | 1 | 1 | 0 | 6 | Medium |
| 23 | Sayasone, 2007 | 1 | 0 | 0 | 1 | 1 | 1 | 1 | 1 | 3 | 6 | Medium |
| 24 | Phathammavong, 2007 | 1 | 1 | 0 | 1 | 1 | 0 | 0 | 1 | 0 | 5 | Medium |
| 25 | Tungtrongchitr, 2007 | 1 | 0 | 0 | 2 | 2 | 1 | 1 | 1 | 3 | 4 | Medium |
| 26 | Erlanger, 2008 | 1 | 1 | 0 | 1 | 1 | 1 | 1 | 1 | 3 | 7 | Low |
| 27 | Nithikathkul, 2009 | 1 | 0 | 0 | 1 | 1 | 1 | 1 | 1 | 3 | 6 | Medium |
| 28 | Wattanayingcharoenchai, 2011 | 1 | 1 | 0 | 1 | 3 | 1 | 1 | 1 | 3 | 6 | Medium |
| 29 | Sayasone, 2011 | 1 | 2 | 0 | 1 | 1 | 1 | 1 | 1 | 0 | 6 | Medium |
| 30 | Wongsawad, 2012 | 0 | 0 | 0 | 0 | 0 | 1 | 1 | 0 | 3 | 2 | High |
| 31 | Kaewpitoon(a), 2012 | 1 | 1 | 0 | 1 | 0 | 1 | 1 | 1 | 1 | 7 | Low |
| 32 | Kaewpitoon(b), 2012 | 1 | 1 | 0 | 1 | 1 | 1 | 1 | 1 | 1 | 8 | Low |
| 33 | Sohn, 2012 | 1 | 2 | 0 | 0 | 0 | 1 | 1 | 1 | 3 | 4 | Medium |
| 34 | Yong, 2012 | 1 | 0 | 0 | 1 | 1 | 1 | 1 | 2 | 3 | 5 | Medium |
| 35 | Saengsawang, 2012 | 1 | 0 | 0 | 1 | 1 | 1 | 1 | 1 | 1 | 7 | Low |
| 36 | Forrer, 2012 | 1 | 1 | 0 | 1 | 1 | 1 | 1 | 1 | 1 | 8 | Low |
| 37 | Songserm, 2012 | 1 | 1 | 0 | 1 | 1 | 1 | 1 | 1 | 3 | 7 | Low |
| 38 | Boonjaraspinyo, 2013 | 1 | 0 | 0 | 1 | 1 | 1 | 1 | 1 | 1 | 7 | Low |
| 39 | Suwannahitatorn, 2013 | 1 | 0 | 0 | 1 | 1 | 1 | 1 | 1 | 1 | 7 | Low |
| 40 | Saengsawang, 2013 | 1 | 0 | 0 | 1 | 0 | 1 | 1 | 1 | 3 | 5 | Medium |
| 41 | Phongluxa, 2013 | 1 | 1 | 0 | 1 | 1 | 1 | 1 | 1 | 1 | 8 | Low |
| 42 | Puttaruk, 2013 | 3 | 0 | 0 | 1 | 0 | 1 | 1 | 1 | 3 | 4 | Medium |
| 43 | Wongsaroj, 2014 | 1 | 3 | 1 | 1 | 0 | 1 | 1 | 1 | 1 | 7 | Low |
| 44 | Miyamoto, 2014 | 1 | 0 | 0 | 1 | 1 | 1 | 1 | 1 | 3 | 6 | Medium |
| 45 | Thaewnongiew, 2014 | 1 | 1 | 0 | 1 | 1 | 1 | 1 | 1 | 1 | 8 | Low |
| 46 | Wiriyakijja, 2014 | 1 | 1 | 1 | 1 | 1 | 1 | 1 | 1 | 1 | 9 | Low |
| 47 | Chaiputcha, 2015 | 1 | 1 | 1 | 1 | 1 | 1 | 1 | 1 | 1 | 9 | Low |
| 48 | Vonghachack, 2015 | 1 | 1 | 0 | 1 | 1 | 1 | 1 | 1 | 3 | 7 | Low |
| 49 | Kaewpitoon, 2015 | 1 | 1 | 1 | 1 | 1 | 1 | 1 | 1 | 1 | 9 | Low |
| 50 | Sato, 2015 | 1 | 0 | 0 | 1 | 1 | 1 | 1 | 1 | 0 | 6 | Medium |
| 51 | Sayasone, 2015 | 1 | 1 | 0 | 1 | 0 | 1 | 1 | 1 | 0 | 6 | Medium |
| 52 | Rujirakul, 2015 | 1 | 0 | 0 | 0 | 0 | 1 | 1 | 1 | 0 | 4 | Medium |
| 53 | Moore, 2015 | 1 | 0 | 0 | 1 | 1 | 1 | 1 | 1 | 0 | 6 | Medium |
| 54 | Bless, 2015 | 1 | 0 | 0 | 1 | 1 | 1 | 1 | 1 | 1 | 7 | Low |
| 55 | Yospanya, 2015 | 1 | 1 | 1 | 1 | 1 | 1 | 1 | 1 | 1 | 9 | Low |
| 56 | Kaewpitoon(a), 2016 | 1 | 1 | 0 | 1 | 1 | 1 | 1 | 1 | 1 | 8 | Low |
| 57 | Saiyachak, 2016 | 1 | 1 | 1 | 1 | 1 | 1 | 1 | 1 | 1 | 9 | Low |
| 58 | Kaewpitoon(b), 2016 | 1 | 1 | 1 | 1 | 1 | 1 | 1 | 1 | 1 | 9 | Low |
| 59 | Kaewpitoon(c), 2016 | 1 | 1 | 2 | 1 | 1 | 1 | 1 | 1 | 2 | 7 | Low |
| 60 | Saengsawang, 2016 | 1 | 1 | 2 | 1 | 0 | 1 | 1 | 1 | 0 | 6 | Medium |
| 61 | Kaewpitoon(d), 2016 | 1 | 1 | 2 | 1 | 0 | 1 | 1 | 1 | 1 | 7 | Low |
| 62 | Kaewpitoon(e), 2016 | 1 | 1 | 0 | 1 | 1 | 1 | 1 | 1 | 0 | 7 | Low |
| 63 | Doi, 2016 | 1 | 1 | 0 | 0 | 0 | 1 | 1 | 1 | 0 | 5 | Medium |
| 64 | Chuangchaiya, 2016 | 1 | 2 | 2 | 1 | 1 | 0 | 0 | 1 | 2 | 4 | Medium |
| 65 | Jamjane, 2016 | 1 | 1 | 0 | 1 | 1 | 1 | 1 | 1 | 3 | 7 | Low |
| 66 | Taepongsorat, 2016 | 1 | 1 | 0 | 1 | 1 | 1 | 1 | 1 | 3 | 7 | Low |
| 67 | Prakobwong, 2017 | 1 | 2 | 1 | 1 | 1 | 1 | 1 | 1 | 1 | 8 | Low |
| 68 | Evdokimov, 2017 | 1 | 1 | 1 | 1 | 1 | 1 | 1 | 1 | 1 | 9 | Low |
| 69 | Kitvatanachai, 2017 | 1 | 1 | 1 | 2 | 2 | 1 | 1 | 1 | 1 | 7 | Low |
| 70 | Aung, 2017 | 1 | 1 | 1 | 1 | 1 | 1 | 1 | 1 | 1 | 9 | Low |
| 71 | Vonghachack(a), 2017 | 1 | 1 | 1 | 1 | 1 | 1 | 1 | 1 | 1 | 9 | Low |
| 72 | Saenna, 2017 | 1 | 2 | 0 | 1 | 1 | 1 | 1 | 1 | 1 | 7 | Low |
| 73 | Vonghachack(b), 2017 | 1 | 1 | 1 | 1 | 1 | 1 | 1 | 1 | 1 | 9 | Low |
| 74 | Wanpinyocheep, 2017 | 1 | 1 | 0 | 1 | 3 | 1 | 1 | 1 | 1 | 7 | Low |
| 75 | Kaewpitoon(a), 2018 | 1 | 0 | 0 | 1 | 1 | 1 | 1 | 1 | 0 | 6 | Medium |
| 76 | Nakbun, 2018 | 1 | 1 | 1 | 1 | 1 | 1 | 1 | 1 | 1 | 9 | Low |
| 77 | Kaewpitoon(b), 2018 | 1 | 0 | 0 | 1 | 1 | 1 | 1 | 1 | 3 | 6 | Medium |
| 78 | Assavapongpaiboon, 2018 | 1 | 1 | 0 | 1 | 1 | 1 | 1 | 1 | 0 | 7 | Low |
| 79 | Kaewpitoon(c), 2018 | 1 | 0 | 0 | 1 | 1 | 1 | 1 | 1 | 0 | 6 | Medium |
| 80 | Laoraksawong, 2018 | 1 | 1 | 1 | 1 | 1 | 1 | 1 | 1 | 0 | 8 | Low |
| 81 | Panithanang, 2018 | 1 | 0 | 0 | 1 | 1 | 1 | 1 | 2 | 0 | 5 | Medium |
| 82 | Thaewnongiew, 2018 | 1 | 1 | 0 | 1 | 0 | 1 | 1 | 1 | 0 | 6 | Medium |
| 83 | Sornlorm, 2019 | 1 | 1 | 0 | 1 | 1 | 1 | 1 | 1 | 0 | 7 | Low |
| 84 | Khieu, 2019 | 1 | 0 | 0 | 0 | 0 | 0 | 0 | 1 | 0 | 2 | High |
| 85 | Sohn, 2019 | 1 | 0 | 0 | 1 | 0 | 1 | 1 | 0 | 0 | 4 | Medium |
| 86 | Thinkhamrop, 2019 | 1 | 1 | 0 | 1 | 1 | 1 | 1 | 1 | 1 | 8 | Low |
| 87 | Chuangchaiya, 2019 | 1 | 1 | 1 | 1 | 1 | 1 | 1 | 1 | 1 | 9 | Low |
| 88 | Sayasone, 2019 | 1 | 1 | 0 | 1 | 1 | 1 | 1 | 1 | 0 | 7 | Low |
| 89 | Rangsin, 2019 | 1 | 0 | 0 | 1 | 1 | 1 | 1 | 1 | 3 | 6 | Medium |
| 90 | Kaewpitoon, 2019 | 1 | 0 | 0 | 1 | 1 | 1 | 1 | 1 | 3 | 6 | Medium |
| 91 | Yoshida, 2019 | 1 | 1 | 1 | 1 | 1 | 1 | 1 | 1 | 1 | 9 | Low |
| 92 | Namwong, 2019 | 1 | 1 | 0 | 0 | 0 | 1 | 1 | 1 | 0 | 5 | Medium |
| 93 | Nak-ai, 2019 | 1 | 1 | 1 | 1 | 1 | 1 | 1 | 1 | 1 | 9 | Low |
| 94 | Boondit, 2020 | 1 | 1 | 0 | 1 | 1 | 1 | 1 | 1 | 1 | 8 | Low |
| 95 | Chuangchaiya, 2020 | 1 | 1 | 1 | 1 | 0 | 1 | 1 | 1 | 1 | 8 | Low |
| 96 | Rattanapitoon, 2020 | 1 | 0 | 0 | 1 | 1 | 1 | 1 | 1 | 1 | 7 | Low |
| 97 | Homsana, 2020 | 1 | 0 | 0 | 1 | 1 | 1 | 1 | 1 | 3 | 6 | Medium |
| 98 | Buathong, 2020 | 1 | 0 | 0 | 0 | 0 | 1 | 1 | 1 | 0 | 4 | Medium |
| 99 | Prakobwong,2020 | 1 | 1 | 0 | 1 | 1 | 1 | 1 | 1 | 1 | 8 | Low |
| 100 | Nak-ai, 2020 | 1 | 1 | 1 | 1 | 1 | 1 | 1 | 1 | 1 | 9 | Low |
| 101 | Wattanawong, 2021 | 1 | 1 | 1 | 1 | 1 | 1 | 1 | 1 | 1 | 9 | Low |
| 102 | Soncharoen, 2021 | 1 | 1 | 1 | 1 | 1 | 1 | 1 | 1 | 1 | 9 | Low |
| 103 | Srithai, 2021 | 1 | 1 | 1 | 1 | 0 | 1 | 1 | 1 | 1 | 8 | Low |
| 104 | Kitphati, 2021 | 1 | 1 | 1 | 1 | 0 | 1 | 1 | 1 | 1 | 8 | Low |
| 105 | Sungkhabut, 2021 | 1 | 1 | 1 | 1 | 0 | 1 | 1 | 1 | 3 | 7 | Low |
| 106 | Kopolrat, 2021 | 1 | 1 | 1 | 1 | 1 | 1 | 1 | 1 | 1 | 9 | Low |
| 107 | Soncharoen, 2022 | 1 | 1 | 1 | 1 | 1 | 1 | 1 | 1 | 1 | 9 | Low |
| 108 | La, 2022 | 1 | 1 | 1 | 1 | 1 | 1 | 1 | 1 | 1 | 9 | Low |
| 109 | Sangkaeo, 2022 | 1 | 1 | 2 | 0 | 0 | 1 | 1 | 1 | 2 | 5 | Medium |
| 110 | Nantapulsab, 2022 | 1 | 1 | 1 | 1 | 1 | 1 | 1 | 1 | 0 | 8 | Low |
| 111 | Boonkasem, 2022 | 1 | 0 | 0 | 1 | 0 | 1 | 1 | 1 | 0 | 5 | Medium |
| 112 | Boonjaraspinyo, 2023 | 1 | 1 | 1 | 1 | 0 | 1 | 1 | 1 | 1 | 8 | Low |
| 113 | Sato, 2010 | 1 | 0 | 0 | 0 | 0 | 1 | 1 | 1 | 0 | 4 | Medium |
| 114 | Laymanivong, 2016 | 1 | 1 | 0 | 1 | 0 | 1 | 1 | 0 | 0 | 5 | Medium |
| 115 | Nakamura, 2017 | 1 | 1 | 0 | 1 | 1 | 1 | 1 | 2 | 2 | 6 | Medium |
| 116 | Chai, 1998 | 1 | 0 | 0 | 0 | 0 | 1 | 1 | 2 | 0 | 3 | High |
| 117 | Htun, 2018 | 1 | 1 | 1 | 1 | 1 | 1 | 1 | 1 | 2 | 8 | Low |
| 118 | Niamnuy, 2016 | 1 | 1 | 0 | 1 | 1 | 0 | 1 | 1 | 0 | 6 | Medium |
| 119 | Ayé, 2015 | 1 | 1 | 2 | 1 | 1 | 1 | 1 | 1 | 2 | 7 | Low |
| 120 | Giboda, 1991 | 1 | 0 | 0 | 1 | 0 | 1 | 1 | 3 | 0 | 4 | Medium |
| 121 | Rim, 2003 | 1 | 1 | 1 | 1 | 1 | 1 | 1 | 1 | 2 | 8 | Low |
| 122 | Dao, 2016 | 1 | 1 | 1 | 1 | 1 | 1 | 1 | 1 | 1 | 9 | Low |
| 123 | De, 2003 | 1 | 0 | 0 | 0 | 0 | 1 | 0 | 0 | 0 | 2 | High |
| 124 | Songserm, 2021 | 1 | 1 | 1 | 1 | 1 | 1 | 1 | 1 | 1 | 9 | Low |
| 125 | Kaewpitoon(a), 2015 | 1 | 1 | 0 | 1 | 1 | 1 | 1 | 1 | 0 | 7 | Low |

**Supplementary Figure S2 – Risk of Bias of *Clonorchis sinensis***


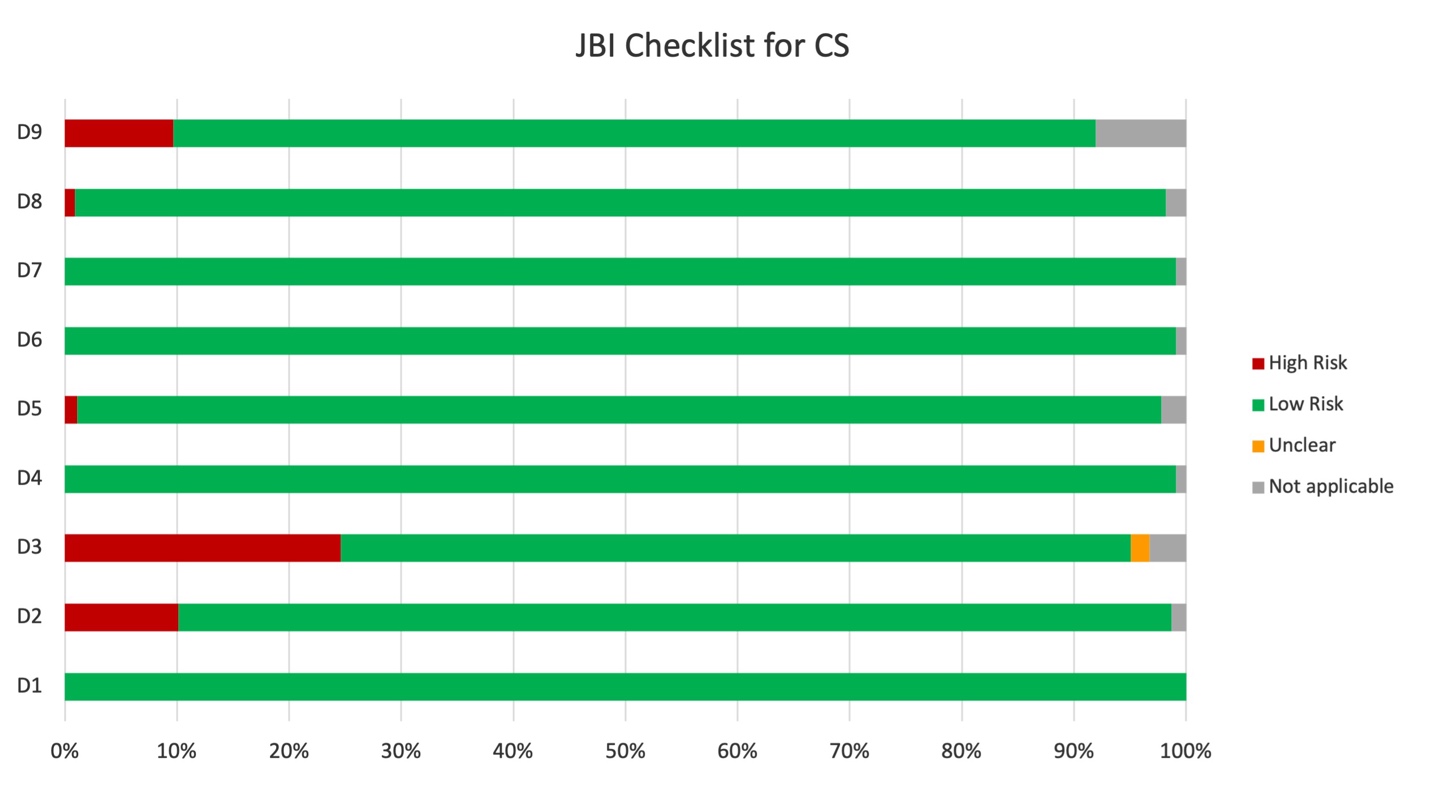


| **Questions:** | |
| --- | --- |
| D1: Was the sample frame appropriate to address the target population? | |
| D2: Were study participants sampled in an appropriate way? | |
| D3: Was the sample size adequate? | |
| D4: Were the study subjects and the setting described in detail? | |
| D5: Was the data analysis conducted with sufficient coverage of the identified sample? | |
| D6: Were valid methods used for the identification of the condition? | |
| D7: Was the condition measured in a standard, reliable way for all participants? | |
| D8: Was there appropriate statistical analysis? | |
| D9: Was the response rate adequate, and if not, was the low response rate managed appropriately? | |
| **Categorical labels:** |  |
| 0= No= High risk, |  |
| 1= Yes= Low risk, |  |
| 2= Un= Unclear |  |
| 3=NA= Not applicable |  |

**Table S3: Risk of bias assessment of included studies for *Clonorchis sinensis* by JBI critical appraisal checklist for studies reporting prevalence data.**

| Article | First Author, Publication Year | D1 | D2 | D3 | D4 | D5 | D6 | D7 | D8 | D9 | Total | QA grade |
| --- | --- | --- | --- | --- | --- | --- | --- | --- | --- | --- | --- | --- |
| 1 | Kino, 1998 | 1 | 1 | 0 | 1 | 1 | 1 | 1 | 1 | 0 | 7 | Low |
| 2 | Hong, 2001 | 1 | 1 | 0 | 1 | 1 | 1 | 1 | 0 | 3 | 6 | Medium |
| 3 | Yu, 2003 | 1 | 1 | 3 | 1 | 3 | 1 | 1 | 3 | 3 | 5 | Medium |
| 4 | Nontasut, 2003 | 1 | 0 | 0 | 1 | 1 | 1 | 1 | 1 | 0 | 6 | Medium |
| 5 | Verle, 2003 | 1 | 1 | 0 | 1 | 1 | 1 | 1 | 1 | 0 | 7 | Low |
| 6 | Dung, 2007 | 1 | 1 | 0 | 1 | 1 | 1 | 1 | 1 | 0 | 7 | Low |
| 7 | Cam, 2008 | 1 | 1 | 0 | 1 | 1 | 1 | 1 | 1 | 0 | 7 | Low |
| 8 | Yajima, 2009 | 1 | 1 | 0 | 1 | 0 | 1 | 1 | 1 | 0 | 6 | Medium |
| 9 | Li, 2011 | 1 | 1 | 1 | 1 | 1 | 1 | 1 | 1 | 1 | 9 | Low |
| 10 | Dan, 2012 | 1 | 1 | 1 | 1 | 1 | 1 | 1 | 1 | 1 | 9 | Low |
| 11 | Jeon, 2012 | 1 | 0 | 0 | 3 | 3 | 3 | 3 | 1 | 3 | 2 | High |
| 12 | Qian, 2014 | 1 | 0 | 0 | 1 | 1 | 1 | 1 | 1 | 1 | 7 | Low |
| 13 | Lai, 2016 | 1 | 0 | 0 | 1 | 1 | 1 | 1 | 3 | 3 | 5 | Medium |
| 14 | Vinh, 2017 | 1 | 1 | 0 | 1 | 1 | 1 | 1 | 1 | 1 | 8 | Low |
| 15 | Zhu, 2020 | 1 | 1 | 1 | 1 | 1 | 1 | 1 | 1 | 1 | 9 | Low |
| 16 | Sun, 2020 | 1 | 1 | 2 | 1 | 1 | 1 | 1 | 1 | 1 | 8 | Low |
| 17 | Nguyen, 2020 | 1 | 1 | 1 | 1 | 1 | 1 | 1 | 1 | 1 | 9 | Low |
| 18 | Xin,2021 | 1 | 1 | 0 | 1 | 1 | 1 | 1 | 1 | 3 | 7 | Low |
| 19 | Jiang, 2021 | 1 | 1 | 0 | 1 | 1 | 1 | 1 | 1 | 1 | 8 | Low |
| 20 | Qian, 2021 | 1 | 3 | 3 | 1 | 1 | 1 | 1 | 1 | 1 | 7 | Low |
| 21 | Xu, 2021 | 1 | 0 | 1 | 1 | 1 | 1 | 1 | 1 | 1 | 8 | Low |
| 22 | Qian, 2022 | 1 | 0 | 0 | 1 | 1 | 1 | 1 | 1 | 1 | 7 | Low |
| 23 | Liu, 2015 | 1 | 0 | 0 | 1 | 1 | 1 | 1 | 1 | 1 | 7 | Low |
| 24 | Fang, 2008 | 1 | 0 | 1 | 1 | 1 | 1 | 1 | 1 | 1 | 8 | Low |
| 25 | LÜ, 2021 | 1 | 1 | 1 | 1 | 1 | 1 | 1 | 1 | 1 | 9 | Low |
| 26 | Li, 2022 | 1 | 1 | 1 | 1 | 1 | 1 | 1 | 1 | 1 | 9 | Low |

**Supplementary Figure S3 Funnel plot of *Opisthorchis viverrini***


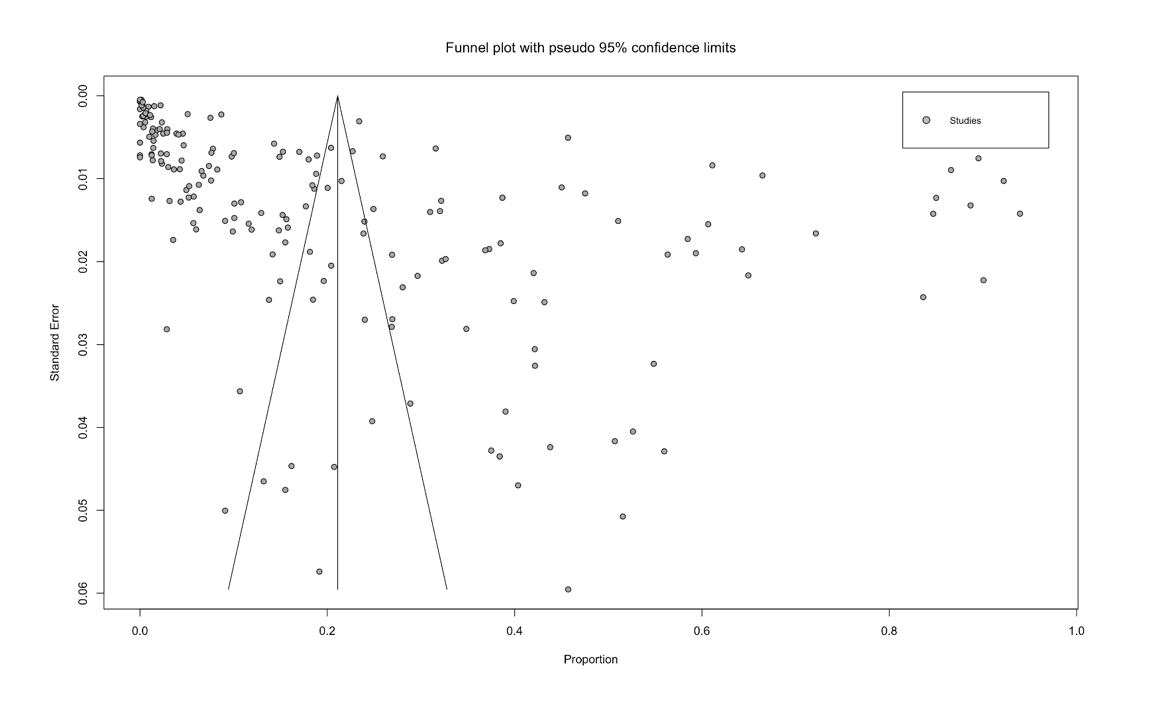


**Figure S3** *Opisthorchis viverrini* studies funnel plot with pseudo 95% confidence *intervals. To* assess publication bias, the *Opisthorchis viverrini* data set was used. The *P*-value of 0.001 determined by Egger's test indicates publication bias.

**Supplementary Figure S4 Funnel plot of *Clonorchis sinensis***


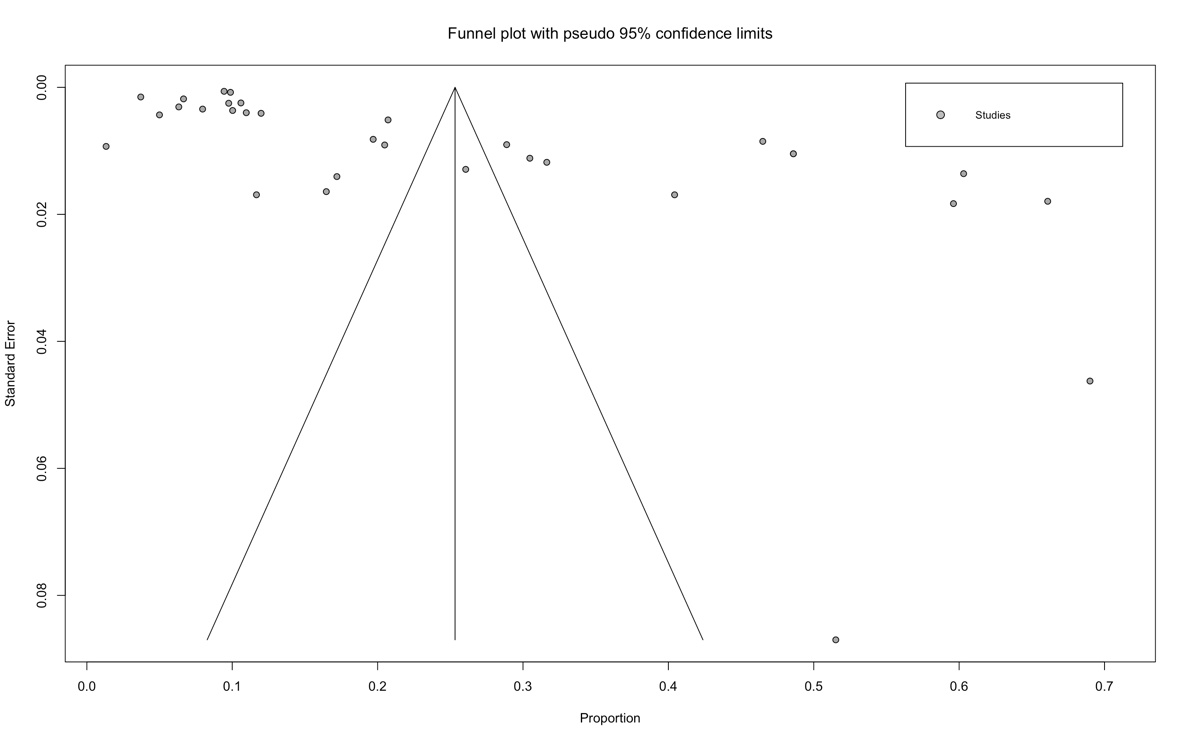


**Figure S4** Clonorchis sinensis studies funnel plot with pseudo 95% confidence intervals. To assess publication bias, the Clonorchis sinensis data set was used. The P-value of 0.006 determined by Egger's test indicates publication bias.

**Supplementary Figures S5, S6, S7, S8 and S9 – Random-effects meta-regression analyses**

***Opisthorchis viverrini***

**
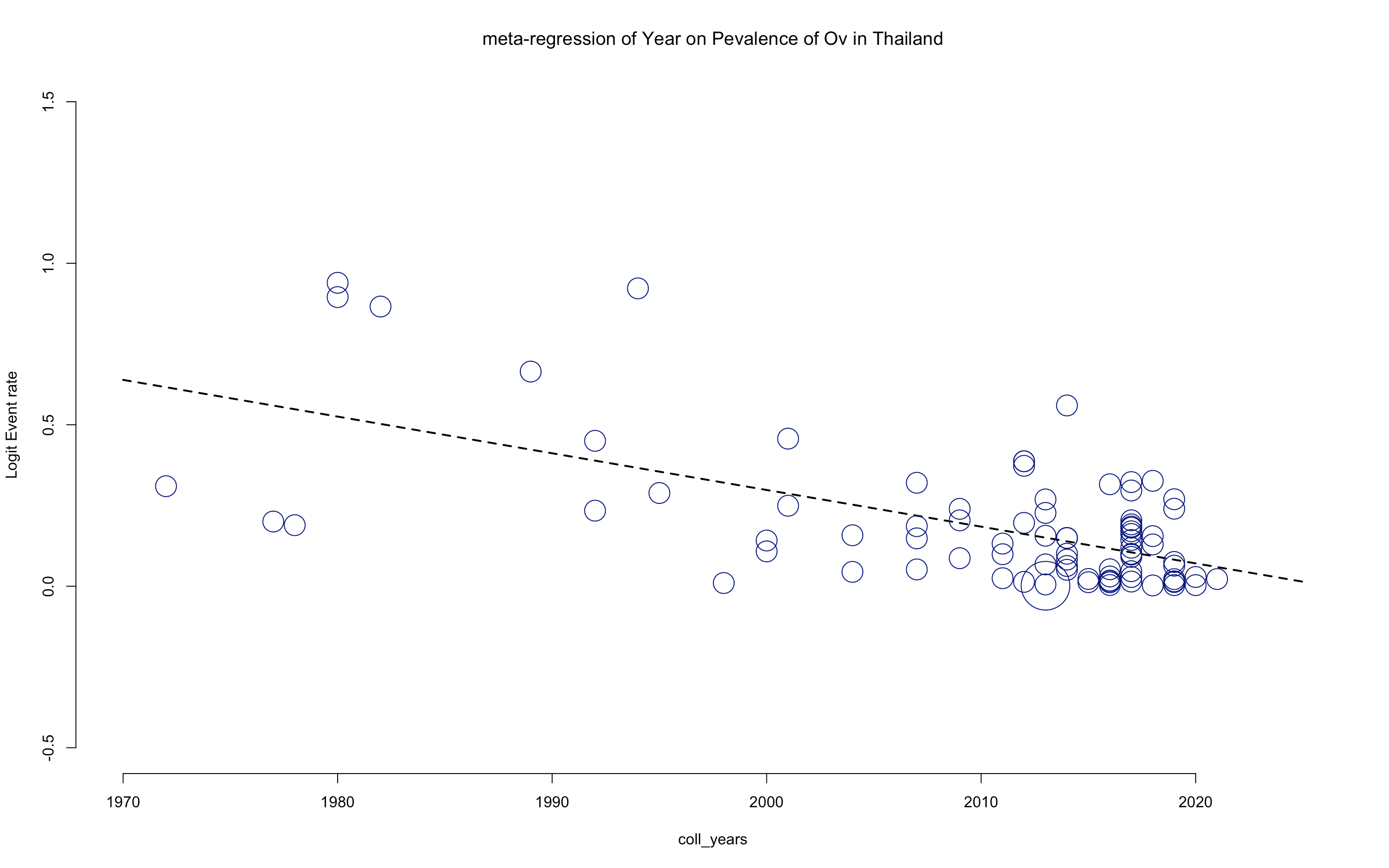
**

**Figure S5** Random-effects meta-regression analyses of the prevalence of Opisthorchis viverrini infection in Thailand by collection year demonstrate a statistically significant downward trend in prevalence.

**
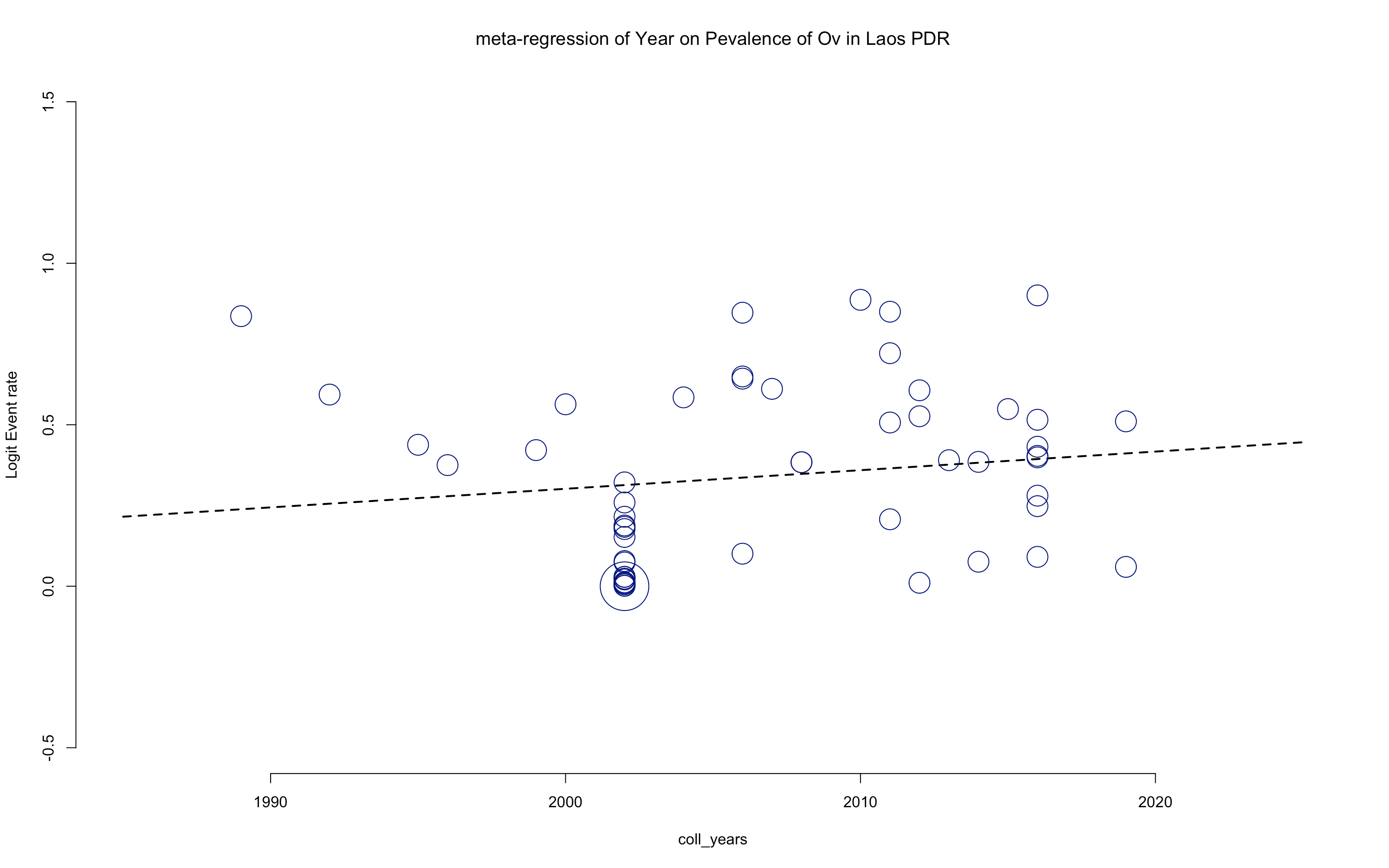
**

**Figure S6** Random-effects meta-regression analyses of the prevalence of Opisthorchis viverrini infection in Laos PDR by collection year demonstrate a statistically non- significant upward trend in prevalence.


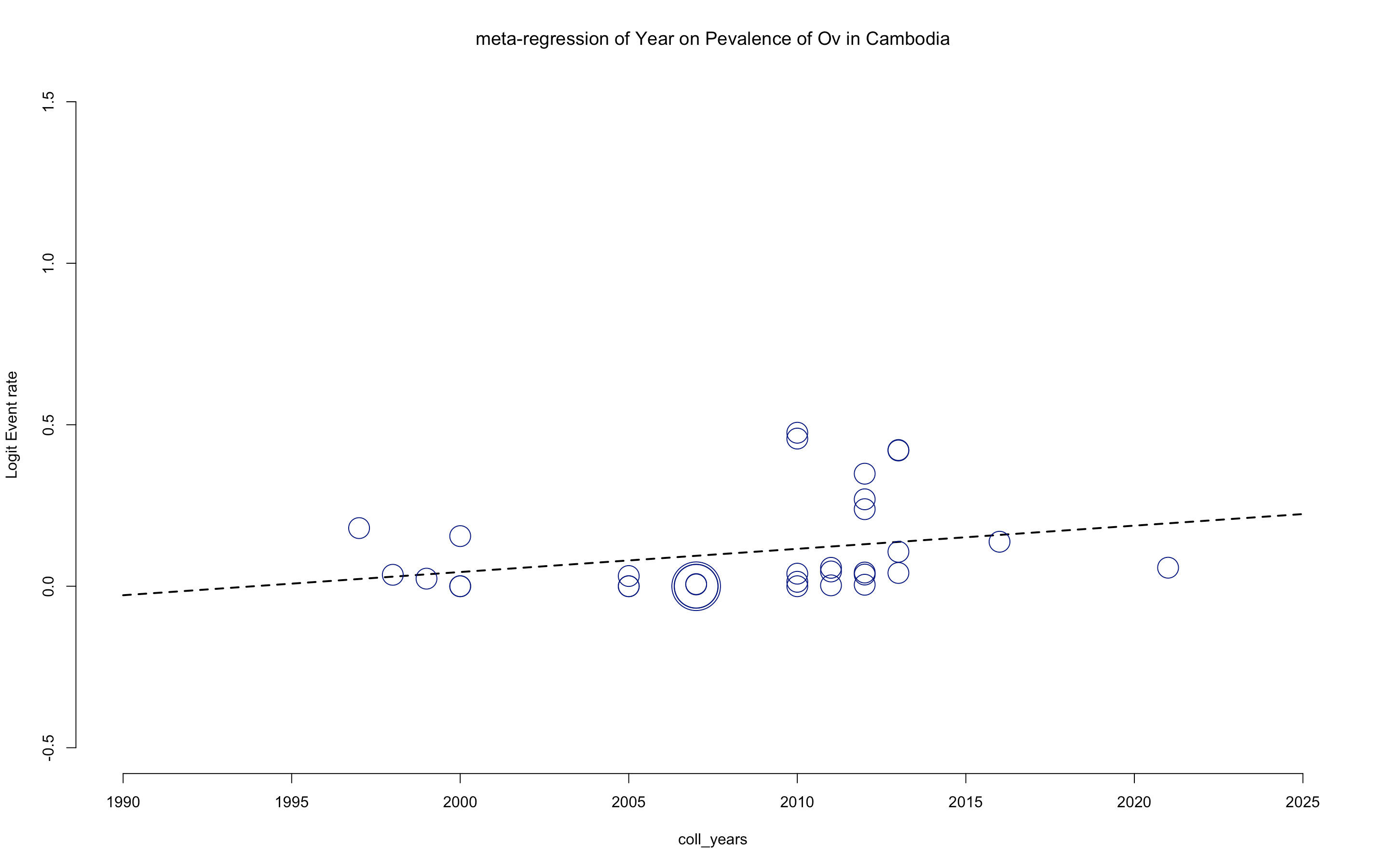


**Figure S7** Random-effects meta-regression analyses of the prevalence of Opisthorchis viverrini infection in Cambodia by collection year demonstrate a statistically non- significant upward trend in prevalence.

***Clonorchis sinensis***


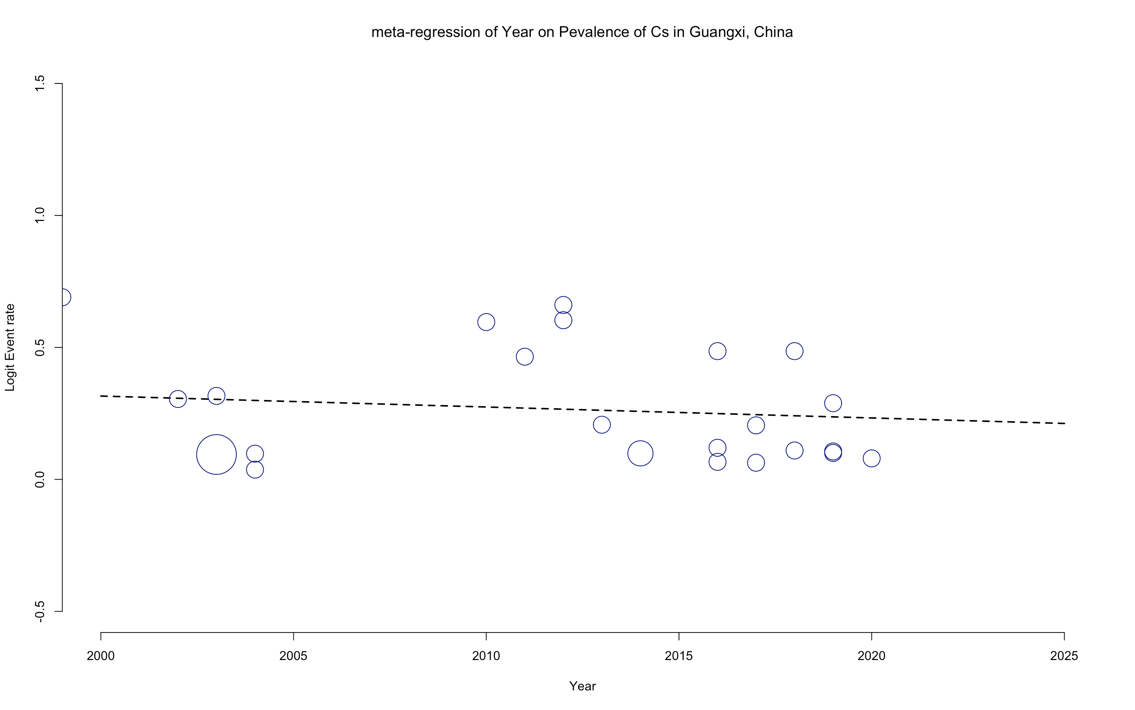


**Figure S8** Random-effects meta-regression analysis of the prevalence of Clonorchis sinensis infection in Guangxi, China by collecting year show a statistically non-significant slightly downward trend in prevalence.


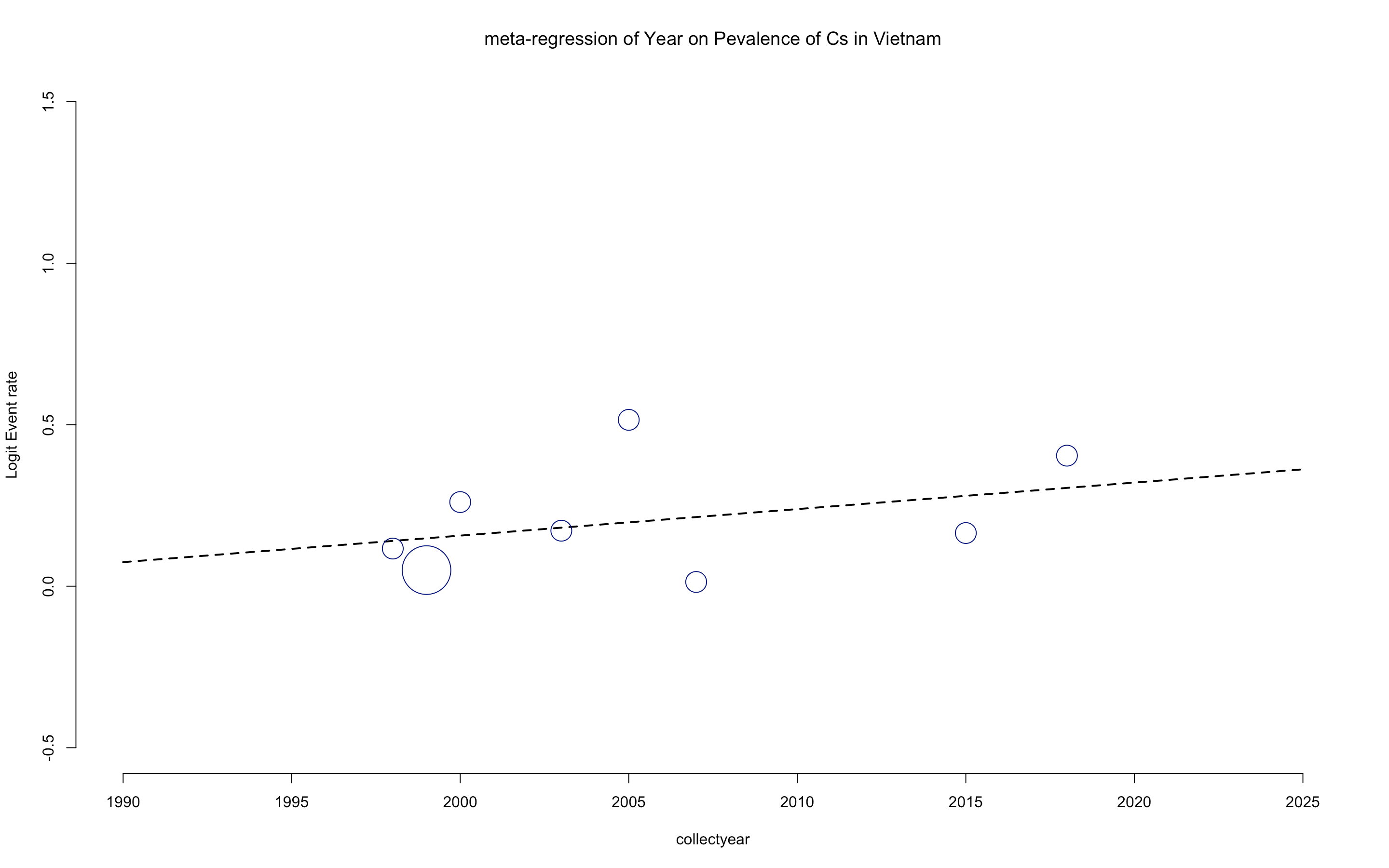


**Figure S9** Random-effects meta-regression analysis of the prevalence of Clonorchis sinensis infection in Vietnam by collecting year show a statistically non-significant increase trend in prevalence.
